# Rate constants of dichloride radical anion reactions with molecules of environmental interest in aqueous solution: a review

**DOI:** 10.1007/s11356-021-14453-w

**Published:** 2021-06-04

**Authors:** László Wojnárovits, Erzsébet Takács

**Affiliations:** Radiation Chemistry Department, Institute for Energy Security and Environmental Safety, Centre for Energy Research, H-1121 Konkoly-Thege Miklós út, Budapest, 29-33 Hungary

**Keywords:** One-electron oxidation, Electron transfer, Pollutant degradation, Advanced oxidation, Water treatment, Toxic organic impurities

## Abstract

Natural waters, water droplets in the air at coastal regions and wastewaters usually contain chloride ions (Cl^-^) in relatively high concentrations in the milimolar range. In the reactions of highly oxidizing radicals (e.g., ^•^OH, ^•^NO_3_, or SO_4_^•-^) in the nature or during wastewater treatment in advanced oxidation processes the chloride ions easily transform to chlorine containing radicals, such as Cl^•^, Cl_2_^•-^, and ClO^•^. This transformation basically affects the degradation of organic molecules. In this review about 400 rate constants of the dichloride radical anion (Cl_2_^•-^) with about 300 organic molecules is discussed together with the reaction mechanisms. The reactions with phenols, anilines, sulfur compounds (with sulfur atom in lower oxidation state), and molecules with conjugated electron systems are suggested to take place with electron transfer mechanism. The rate constant is high (10^7^–10^9^ M^-1^ s^-1^) when the reduction potential the one-electron oxidized species/molecule couple is well below that of the Cl_2_^•-^/2Cl^-^ couple.

**Supplementary Information:**

The online version contains supplementary material available at 10.1007/s11356-021-14453-w.

## **Introduction**

The presence of chloride ions in wastewaters is widespread. Therefore, the effect of chloride ions on Advanced Oxidation Processes (AOP), emerging technology for wastewater purification, is of outermost importance since Cl^-^ efficiently scavenges the reacting radicals (hydroxyl radical (^•^OH), sulfate radical anion (SO_4_^•-^)) transforming them to chlorine containing radicals, e.g., chloride atom and dichloride radical anion (^•^Cl and Cl_2_^•-^). However, the latter radicals also react with organic molecules, with altered rate constants and selectivity (Caregnato et al. 2013). In surface waters and treated wastewaters rich in Cl^-^ ions, including oceans, estuaries, and brines chloride radical anions can occur at concentrations that are orders of magnitude higher than the concentrations of other radicals such as ^•^OH. These radicals may also form during disinfection by chlorine or during chlorine photolysis (Zhang and Parker 2018; Liu et al. 2019). Therefore, determination of the reactivity of chloride radicals, among them also that of Cl_2_^•-^ toward organic molecules is of environmental importance.

The standard reduction potential of the Cl_2_^•-^/2Cl^-^ couple has been reported to be between 2.1 and 2.3 V *vs*. NHE (Wardman 1989; Armstrong et al. 2015), here we use the frequently referred value: *E*^o^(Cl_2_^•-^/2Cl^-^) = 2.1 V. We often compare the rate constants measured in Cl_2_^•-^ reactions with values measured in reactions of other one-electron oxidants. Comparison of the reduction potentials of the one-electron oxidants and those of the semioxidized and nonoxidized forms of organic molecule couples often gives possibilities for explaining the rate constant ranges of radical reactions. Table 1S in the Supplementary material lists several one-electron oxidants with reduction potentials ranging from 2.43 to 0.934 V.

**Table 1:** Nonaromatic organic molecules

Compound, p*K*_a_	*k*_Cl2•-_, M^-1^ s^-1^	Method, pH	Literature
*Molecules with interest from tropospheric point of view*			
Methanol	3.5±0.7 × 10^3^	Pr, 1	Hasegawa and Neta (1978)
	4 × 10^3^	Pr	Feldman et al. (1986)
	5.1±0.3 × 10^4^	LFP, 4	Jacobi et al. (1999)
	2.9±0.8 × 10^4^	LFP, 4	
Ethanol	4.5±0.9 × 10^4^	Pr, 1	Hasegawa and Neta (1978)
	1.4 × 10^4^	LFP	Khmelinskii et al. (1989)
	1.2±0.2 × 10^5^	Pr, 4	Jacobi et al. (1999)
	1.77±0.37 × 10^5^	LFP	Strekowski et al. (2003)
1-Propanol	1.01±0.07 × 10^4^	Pr, 4	Jacobi et al. (1999)
2-Propanol	1.9 × 10^5^	Pr, ~0.3	Storer et al. (1975)
	1.2±0.2 × 10^5^	Pr, 1	Hasegawa and Neta (1978)
	1.5 × 10^5^	FP, 1	Henglein (1982)
	1.9±0.3 × 10^5^	LFP, 4	Jacobi et al. (1999)
*tert*-Butanol	~7 × 10^2^	Pr, 1	Hasegawa and Neta (1978)
	2.6±0.5 × 10^4^	LFP, 4	Jacobi et al. (1999)
Diethylether	4.0±0.2 × 10^5^	LFP, 4	Jacobi et al. (1999)
Methyl-*tert*-butylether	7±1 × 10^4^	LFP, 4	Jacobi et al. (1999)
Tetrahydrofuran	4.8±0.6 × 10^5^	LFP, 4	Jacobi et al. (1999)
	5.3±0.6 × 10^6^	LFP, 4	Wicktor et al. (2003)
1,4-Dioxan	3.3 × 10^6^		Patton et al. (2016)
Acetone	1.4±0.3 × 10^3^	Pr, 1	Hasegawa and Neta (1978)
	1.8 × 10^3^	Pr	Broszkiewicz et al. (1981)
	1.41±0.09 × 10^3^	LFP, 4	Jacobi et al. (1999)
Formaldehyde	3.6±0.5 × 10^4^	LFP, 4	Jacobi et al. (1999)
Formic acid, 3.77	6.7±1.3 × 10^3^	Pr, 1	Hasegawa and Neta (1978)
	3±1 × 10^5^	Pr, 3	
	3.7±1.0 × 10^6^	Pr, 3.7	
	1.9±0.4 × 10^6^	Pr, 7	
	8.0±1.4 × 10^4^	LFP, 0	Jacobi et al. (1999)
Acetic acid, 4.7	<10^4^	Pr, 1	Hasegawa and Neta (1978)
	1.4±0.2 × 10^6^	FP, 6–7	Masuda et al. (1979)
	1.5±0.8 × 10^3^	LFP, 0.4	Jacobi et al. (1999)
Propionic acid, 4.88	2.2±0.5 × 10^3^	Pr, 1	Hasegawa and Neta (1978)
	2.5±0.5 × 10^6^	FP, 6–7	Masuda et al. (1979)
Succinic acid, 4.2, 5.6	~8 × 10^2^	Pr, 1	Hasegawa and Neta (1978)
Acetylacetone, 8.9	1.42±0.08 × 10^8^	LFP, 7	Lei et al. (2019)
α-Pinene	1.7 × 10^8^	Pr	Kluge et al. (1998)
*Unsaturated alcohols and carboxylic acids*			
Allyl alcohol	5.9 × 10^8^	Pr, 1	Hasegawa and Neta (1978)
	5.9 × 10^8^	Pr, 7	Hasegawa and Neta (1978)
	5.0 × 10^7^	LFP	Padmaja et al. (1992)
	8.5 × 10^7^	Pr, 2.3	Padmaja et al. (1992)
	5.2 × 10^7^	LFP	Alfassi et al. (1993)
2-Buten-1-ol	1.8 × 10^8^	LFP	Padmaja et al. (1992)
	1.9 × 10^8^	LFP	Alfassi et al. (1993)
3-Buten-1-ol	9.9 × 10^7^	LFP	Padmaja et al. (1992)
3-Buten-2-ol	5.2 × 10^7^	LFP	Padmaja et al. (1992)
4-Penten-2-ol	9.6 × 10^7^	LFP	Padmaja et al. (1992)
2-Methyl-2-propen-1-ol	3.1 × 10^8^	LFP	Padmaja et al. (1992)
3-Methyl-3-buten-1-ol	4.7 × 10^8^	LFP	Padmaja et al. (1992)
3-Methyl-2-buten-1-ol	7.0 × 10^8^	LFP	Padmaja et al. (1992)
2-Cyclohexen-1-ol	1.7 × 10^8^	LFP	Padmaja et al. (1992)
Fumaric acid	~2 × 10^5^	Pr, 1	Hasegawa and Neta (1978)
	1.2±1.0 × 10^5^	Pr, 1.5	Wojnárovits et al. (2008)
Fumaric acid, monoanion	2.4 × 10^6^	Pr, 3.7	Hasegawa and Neta (1978)
	2.0±0.3 × 10^6^	Pr, 3.5	Wojnárovits et al. (2008)
Fumaric acid, dianion	4 × 10^6^	Pr, 7	Hasegawa and Neta (1978)
	4.5±0.3 × 10^6^	Pr, 6	Wojnárovits et al. (2008)
Maleic acid	1.7±0.2 × 10^6^	Pr, 1.5	Wojnárovits et al. (2008)
Maleic acid, monoanion	1.25±0.1 × 10^6^	Pr, 4	Wojnárovits et al. (2008)
Maleic acid, dianion	3 × 10^6^	Pr, 6.5	Hasegawa and Neta (1978)
	3.4±0.1 × 10^6^	Pr, 7.5	Wojnárovits et al. (2008)
Acrylic acid, 4.25	5.4 × 10^6^	Pr, 1	Hasegawa and Neta (1978)
	1.9 × 10^7^	Pr, 7	Hasegawa and Neta (1978)
n-Butyl acrylate	2.4 × 10^6^	Pr, 2	Sabharwal et al. (1998)
3-Sulfo-propylmethacrylate	3.5 × 10^7^	Pr	Panda et al. (2001)
Acrylamide	6.6 × 10^6^	LFP	Padmaja et al. (1992)
N-Isopropyl acrylamide	2 × 10^7^	Pr, 1	Acharya et al. (2003)
Acrylonitrile	2.2 × 10^6^	Pr, 7	Hasegawa and Neta (1978)
Muconic acid, 3.87	2.1 × 10^8^	Pr, 7	Hasegawa and Neta (1978)
Sorbic acid, 4.76	6.8 × 10^8^	Pr, 7	Hasegawa and Neta (1978)
3-Hexenedioic acid, 3.92	1.6 × 10^8^	Pr, 7	Hasegawa and Neta (1978)
*Small organic molecules containing halogen atoms*			
Monofluoroacetic acid, 2.59	2.2±0.2 × 10^5^	LFP, 4–5	Maruthamuthu et al. (1995)
Difluoroacetic acid, 1.33	<10^4^	LFP, 4–5	Maruthamuthu et al. (1995)
Trifluoroacetic acid, 0.23	<10^4^	LFP, 4–5	Maruthamuthu et al. (1995)
Monochloroacetic acid, 2.87	6.5±0.1 × 10^4^	LFP, 4–5	Maruthamuthu et al. (1995)
Dichloroacetic acid, 1.35	1.5±0.1 × 10^4^	LFP, 4–5	Maruthamuthu et al. (1995)
Trichloroacetic acid, 0.66	4 × 10^4^	LFP, 4–5	Maruthamuthu et al. (1995)
Trichloroethylene	1 × 10^7^		Li et al. (2007)
1,1,1-Trifluoro-2-iodoethane	2 × 10^7^	Pr	Mohan et al. (1994)
Diiodomethane	1.7 × 10^8^	Pr	Mohan and Moorthy (1990b)

### Determination of the Cl_2_^•-^ reaction rate constants

The bimolecular rate constants of Cl_2_^•-^ reactions published in the literature were mainly determined in pulse radiolysis and (laser) flash photolysis experiments (Neta et al. 1988). In contrast to other oxidizing radicals (e.g., ^•^OH, SO_4_^•-^, and CO_3_^•-^), competitive techniques were rarely used in rate constant determinations (Liu et al. 2020). This is probably due to the rather complex reaction system involved in Cl_2_^•-^ reactions (see below). A reference compound in the solution would make further complications.

In pulse radiolysis Cl_2_^•-^ reactions are generally investigated in N_2_O-saturated solutions Buxton et al. (1988b) at high Cl^-^ concentration relative to the compound of interest (S), where most of ^•^OH are scavenged by Cl^-^, transforming the hydroxyl radical to Cl^•^ and then to Cl_2_^•-^ (Hasegawa and Neta 1978). The pH is adjusted to the acidic range (pH 1–4) to ensure the efficient formation of Cl_2_^•-^. Manifold reactions proceed in such a system (Buxton et al. 1988a, b; Brigante et al. 2014; Szala-Bilnik et al. 2014; Kazmierczak et al. 2015, 2019).

1 $$ {}^{\bullet}\mathrm{OH}+{\mathrm{Cl}}^{-}\rightleftarrows {\mathrm{Cl}\mathrm{OH}}^{\bullet -}{k}_1=4.3\times {10}^9{\mathrm{M}}^{-1}{\mathrm{s}}^{-1} $$

-1 $$ {k}_{-1}=6.1\times {10}^9{\mathrm{s}}^{-1} $$

2 $$ {\mathrm{ClOH}}^{\bullet -}+{\mathrm{H}}^{+}\rightleftarrows {\left(\mathrm{HOClH}\right)}^{\bullet }{k}_2=3\times {10}^{10}{\mathrm{M}}^{-1}{\mathrm{s}}^{-1} $$

−2 $$ {k}_{-2}=1\times {10}^8{\mathrm{s}}^{-1} $$

3 $$ {\left(\mathrm{HOClH}\right)}^{\bullet}\rightleftarrows {\mathrm{Cl}}^{\bullet }+{\mathrm{H}}_2{\mathrm{O}k}_3=\left(5\pm 2\right)\times {10}^4{\mathrm{s}}^{-1} $$

−3 $$ {k}_{-3}=2.5\times {10}^5{\mathrm{s}}^{-1} $$

4 $$ {\mathrm{Cl}}^{\bullet }+{\mathrm{Cl}}^{-}\rightleftarrows {\mathrm{Cl}}_2{k}_4=8.5\pm 0.6\times {10}^9{\mathrm{M}}^{-1}{\mathrm{s}}^{-1} $$

−4 $$ {k}_{-4}=6.0\pm 0.5\times {10}^4{\mathrm{s}}^{-1} $$

$$ K=\frac{k_4}{k_{-4}}=1.4\times {10}^5\ {M}^{-1} $$

5 $$ {{\mathrm{Cl}}_2}^{\bullet -}+{\mathrm{H}}_2\mathrm{O}\rightleftarrows {\left(\mathrm{HOClH}\right)}^{\bullet }+{\mathrm{Cl}}^{-}{k}_5=1300{\mathrm{s}}^{-1} $$

−5 $$ {k}_{-5}=\left(8\pm 2\right)\times {10}^9\ {M}^{-1}\ {\mathrm{s}}^{-1} $$

6 $$ {\mathrm{Cl}}_2^{\kern0.5em \bullet -}+S\to products\ {k}_6\ \left(={k}_{Cl2\bullet -}\right) $$

7 $$ {\mathrm{Cl}}^{\bullet }+S\to products\ {k}_7 $$

**Scheme 1:**
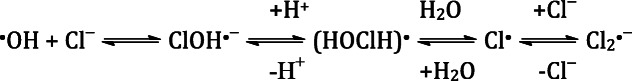
Simplified scheme of Cl_2_^•-^ formation in ^•^OH reaction

^•^OH in fast reaction with Cl^-^ forms ClOH^•-^ complex (Reaction 1), with an equilibrium constant of 0.70 ± 0.13 M^-1^. In acidic media ClOH^•-^ may transform to Cl^•^ in Eq. (3). Cl^•^ is a strong oxidant that can react directly with dissolved organic material (*E*^o^(Cl^•^/Cl^-^) = 2.6 V, Wardman 1989) with rate constants that are 1–5 orders of magnitude higher than those in the reactions of Cl_2_^•-^. However, in rapid complexation Eq. (4) with Cl^-^, Cl^•^ transforms to Cl_2_^•-^. The determination of rate constants of Cl_2_^•-^ reactions requires high Cl^-^ concentrations (0.1-1 M), and ionic strength corrections (De Laat and Stefan 2017). We mention that such corrections were made only in a few works. The complicated reaction/equilibrium system is summarized in the simplified Scheme 1. As this scheme also shows high pH pushes the reaction system in the direction of ^•^OH, whereas low pH is favorable for Cl_2_^•-^. Therefore, in the ^•^OH + Cl^-^ system, at sufficiently high Cl^-^ concentration in the acidic pH range Cl_2_^•-^ dominates, in the alkaline pH range ^•^OH is the main reactant (Yu and Barker 2003; Yu et al. 2004).

Under suitable conditions, when a compound of interest (S) is present in the system, Cl_2_^•-^ decays by first-order kinetics (*k*_obs_). In laboratory experiments, at the usual Cl^-^ concentration, equilibrium Eq. (4) is assumed to be attained. If so, *k*_obs_ can be expressed as Eq. (8) when *k*_4_ [Cl^-^] + *k*_-4_ >> (*k*_6_ + *k*_7_) [S] + *k*_5_ + *k*_-3_ and *K* [Cl^-^] >> 1 (for more details see Buxton et al. 1988a):

8 $$ {k}_{\mathrm{obs}}={k}_6+{k}_{-4}/\left(K\left[{\mathrm{Cl}}^{-}\right]\right)+\left\{\left({k}_7/K\left[{\mathrm{Cl}}^{-}\right]\right)+{k}_6\right\}\left[\mathrm{S}\right] $$

Under the usual conditions *k*_7_/*K* [Cl^-^] << *k*_6_, and Eq. (8) simplifies to *k*_obs =_ const. + *k*_6_[S]. In purified water, in the absence of suitable reactants, Cl_2_^•-^ may disappear from the solution in disproportionation reaction (Kazmierczak et al. 2015, 2019):

9 $$ {{\mathrm{Cl}}_2}^{\bullet -}+{{\mathrm{Cl}}_2}^{\bullet -}---\to {\mathrm{Cl}}_2+2{\mathrm{Cl}}^{-}{k}_9=1.47\times {10}^9{\mathrm{M}}^{-1}{\mathrm{s}}^{-1}\left(\mathrm{zeroionicstrength}\right) $$

In pulse radiolysis mainly the radiolytically formed ^•^OH (Buxton 2008) is used for Cl_2_^•-^ production. In the laser photolysis experiments of Lanzafame et al. (2017) ^•^OH was produced in H_2_O_2_ photodissociation. In their basic work Hasegawa and Neta (1978) in the pH 1–3 range utilized ^•^OH for Cl^-^ oxidation, in experiments above this pH range SO_4_^•-^ was used to initiate Cl_2_^•-^ formation. When the SO_4_^•-^ technique is combined with pulse radiolysis, SO_4_^•-^ forms in the reactions of hydrated electrons (e_aq_^-^) with persulfate anions (S_2_O_8_^2-^):

10 $$ {{\mathrm{e}}_{\mathrm{aq}}}^{-}+{\mathrm{S}}_2{{\mathrm{O}}_8}^{2-}\hbox{---} \to {{\mathrm{S}\mathrm{O}}_4}^{\bullet -}+{{\mathrm{S}\mathrm{O}}_4}^{2-} $$

11 $$ {{\mathrm{SO}}_4}^{\bullet -}+{\mathrm{Cl}}^{-}\rightleftarrows {{{\mathrm{SO}}_4}^2}^{-}+{\mathrm{Cl}}^{\bullet }{k}_{11}=3.2\times {10}^8{\mathrm{M}}^{-1}{\mathrm{s}}^{-1} $$

–11 $$ {k}_{-11}=2.5\times {10}^8\ {\mathrm{M}}^{-1}\ {\mathrm{s}}^{-1} $$

In laser flash photolysis experiments SO_4_^•-^ may be produced in photon induced splitting of S_2_O_8_^2-^ (Jacobi et al. 1999; Mártire et al. 2001; Caregnato et al. 2013).

12 $$ {\mathrm{S}}_2{{\mathrm{O}}_8}^{2-}+ h\nu \hbox{---} \to 2\ {{\mathrm{S}\mathrm{O}}_4}^{\bullet -} $$

Cl_2_^•-^ has a wide transient absorption band with *λ*_max_ = 340 nm and *ε*_340 nm_ = 9600 M^-1^ cm^-1^, at 340 nm the absorbance of Cl^•^ is negligible compared to that of Cl_2_^•-^ (Guha et al. 1992; Caregnato et al. 2013). In transient experiments mostly this band is used for rate constant determination. However, many organic radical intermediates formed in Cl_2_^•-^ reaction, e.g., cyclohexadienyl or phenoxyl radicals, have absorption bands around or slightly above 340 nm. This coincidence complicates the investigation of organic radical intermediates; the mechanistic suggestions are usually based on indirect information. There are exceptions, e.g., certain dye cations (methylene blue, toluidine blue, safranine T, Kishore et al. 1989; Mahadevan et al. 1990; Guha et al. 1992) have strong absorbances out of this range which allow direct observation of product build-up. Transient products were identified only in few cases (e.g., Dwibedy et al. 2005; Osiewala et al. 2013; Caregnato et al. 2013). In mechanistic studies, instead of Cl_2_^•-^, sometimes azide radical (N_3_^•^) is used, this radical does not have light absorption in the 300-500 nm range (Buxton and Janovský 1976; Hug 1981) .

When Cl^•^ is produced in the SO_4_^•-^ + Cl^-^ reaction, the absorbance of SO_4_^•-^ slightly disturbs the observation of the Cl_2_^•-^ intermediate in transient measurements, since SO_4_^•-^ has a wide absorption band in the UV–Vis range, *λ*_max_ = 450 nm. The molar absorbance at 340 nm is 1600±180 M^-1^ cm^-1^ (Yu and Barker 2003). However, at sufficiently high Cl^-^ concentration the intense absorbance of Cl_2_^•-^ builds-up practically during the pulse. Fitting to the decay curve of the 340 nm absorbance supplies the pseudo-first-order rate constants (*k*_obs_). Under suitable conditions the slope of the pseudo-first-order rate constants *vs*. solute concentration plot gives the second order rate constants of the Cl_2_^•-^ + S reaction (*k*_Cl2•-_).

Cl^•^, and through the chlorine atom, Cl_2_^•-^ may also be produced in VUV photolysis of Cl^-^ containing solutions (Takahashi et al. 1985). In steady-state experiments sometimes *k*_Cl2•-_ is determined by fitting to complex kinetic models without applying real competitor. Generally, photon intensities, quantum yields, rate constants of some basic reactions of intermediates and the time dependence of degradation are used for *k*_Cl2•-_ calculation. In some works, very large set of reactions was considered, and modeling software was used for obtaining rate constants.

In this review we evaluate the rate constants and reaction mechanisms of Cl_2_^•-^ reactions with about 300 organic molecules, most of which have some environmental implications either as water pollutant, or as an atmospheric contaminant in water droplets. Although in several publications larger number of rate constants were published (Hasegawa and Neta 1978; Cornelius 1998; Jacobi et al. 1999; Jasper et al. 2016; Lei et al. 2019), only a few works discussed structure effects and compared the reactions induced by several one-electron oxidants, including also Cl_2_^•-^. In the tables we collected the rate constants measured around room temperature, only a few termperature dependence studies are published in the literature. The tables show also the p*K*_a_ values collected from a number of publications, e.g., Babic et al. (2007), Shalaeva et al. (2008). The error bounds represent the σ–level uncertainty published in the original works. The methods of *k*_Cl2•-_ determinations are indicated by the following abbreviations: **PR** pulse radiolysis, **FP** flash photolysis, **LFP** laser flash photolysis, **Comp**. competitive method, and **Complex** calculations involving complicated reaction sequence in steady-state experiments.

## Nonaromatic organic molecules

### Molecules with interest from tropospheric point of view

In tropospheric liquid phase (e.g., in droplets), chloride is an abundant species. In Cl^-^ containing aerosols and smaller cloud droplets Cl_2_^•-^ is expected to be formed in reactions of other highly reactive radicals such as ^•^OH, ^•^NO_3_, or SO_4_^•-^ (Jacobi et al. 1999). Soluble oxygenated compounds, alcohols, aldehydes, terpenes, sulfoxides, etc., represent important classes of tropospheric species (Table 1). They may originate either from the gas-phase oxidation of volatile organic compounds or from direct emission (Herrmann et al. 2000, 2015).

Most rate constants of Cl_2_^•-^ reactions with small oxygen containing molecules (Scheme 1S, Supplementary material) were measured by Hasegawa and Neta (1978) and by Jacobi et al. (1999). Eight of the reactions in Table 1 were investigated by both groups. All values are very small, they are in the in the 10^3^–10^6^ M^-1^ s^-1^ range. In this range the uncertainty in the transient measurements is rather large. However, in some cases (e.g., 2-propanol, acetone) the results of the two groups agree excellently. In other cases the *k*_Cl2•-_ values agree within one order of magnitude. The experiments of Hasegawa and Neta were carried out in the presence of 1 M NaCl, while Jacobi et al. applied one order of magnitude smaller NaCl concentration. The differences between the values determined by the two groups may also be attributed to the different experimental conditions. We suggest accepting the values of the latter authors due to the smaller ionic strength effect. The *k*_Cl2•-_ values show some correlation with the energy of the weakest C-H bonds suggesting that the rate is controlled by the energy of the C-H bond being ruptured. For a HROH molecule Hasegawa and Neta (1978) supposed the reaction in the following way:

13 $$ {{\mathrm{Cl}}_2}^{\bullet -}+\mathrm{HROH}\hbox{---} \to \left[{{\mathrm{H}\mathrm{Cl}}_2}^{-}+{}^{\bullet}\mathrm{ROH}\right]\hbox{---} \to 2\ {\mathrm{Cl}}^{-}+{\mathrm{H}}^{+}+{}^{\bullet}\mathrm{ROH} $$

Jacobi et al. (1999) in cases of compounds with high bond dissociation energy (BDE ≈ 410 kJ mol^-1^) speculated about an addition/elimination mechanism:

14 $$ {{\mathrm{Cl}}_2}^{\bullet -}+\mathrm{HROH}\hbox{---} \to \left[{\mathrm{Cl}}^{-}+{}^{\bullet}\mathrm{Cl}-\mathrm{H}-\mathrm{ROH}\right]\hbox{---} \to 2\ {\mathrm{Cl}}^{-}+{\mathrm{H}}^{+}+{}^{\bullet}\mathrm{ROH} $$

α-Pinene may serve as a representative of terpenes. Terpenes are emitted in the atmosphere in large quantities by both anthropic and natural sources. Radical reactions are suggested to strongly contribute to their degradation in water droplets in air. The rate constants of α-pinene reaction with Cl_2_^•-^, 1.7 × 10^8^ M^-1^ s^-1^ (Kluge et al. 1998), is several orders of magnitude higher than the values determined for the previously discussed small oxygenated molecules. The high *k*_Cl2•-_ is certainly due to the double bond in α-pinene which renders Cl_2_^•-^ addition mechanism possible as it was suggested for reactions with acrylic type molecules (Hasegawa and Neta 1978). The reaction gives chlorinated α-pinene.

### Unsaturated alcohols and carboxylic acids

**Scheme 2:**
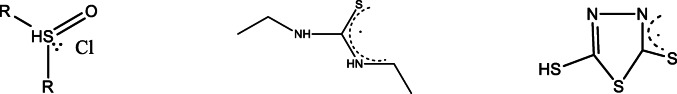
Three-electron bonded systems in one-electron oxidation of dialkylsulfoxides, diethylthiourea and 2,5-dimercaptothiadiazole (Kishore and Asmus 1991; Kishore et al. 1995; Dey et al. 1994c)

Hasegawa and Neta (1978), Padmaja et al. (1992) and Alfassi et al. (1993) published several rate constants on Cl_2_^•-^ reactions with unsaturated alcohols (Scheme 2S). The values increase with the increasing alkyl substitution at the double bond from 5 × 10^7^ to 7 × 10^8^ M^-1^ s^-1^ indicating an electrophile addition mechanism. Cl-adducts were observed by ESR spectroscopy in Cl_2_^•-^ reaction with several compounds.

15 $$ {{\mathrm{C}\mathrm{l}}_2}^{\bullet -}+{\mathrm{R}}_1\mathrm{C}={\mathrm{C}\mathrm{R}}_2---\to \left[{\mathrm{R}}_1{\mathrm{C}}^{\bullet }=\mathrm{C}\left({{\mathrm{C}\mathrm{l}}_2}^{-}\right){\mathrm{R}}_2\right]---\to {\mathrm{C}\mathrm{l}}^{-}+{\mathrm{R}}_1{\mathrm{C}}^{\bullet }=\mathrm{C}\left(\mathrm{Cl}\right){\mathrm{R}}_2 $$

Due to the limited pH range in Cl_2_^•-^ reaction investigations few pH dependence studies were published in the literature. In the practically used pH range Cl_2_^•-^ is a single species (no protonation). Therefore, the changes in the reactivity with the pH must be attributed to the substrate molecules themselves. This is well exemplified by the reactions of maleic and fumaric acids (Hasegawa and Neta 1978; Wojnárovits et al. 2008). These acids undergo protolytic dissociations with p*K*_a1_ = 3.02, p*K*_a2_ = 4.39, and p*K*_a1_ = 1.92, p*K*_a2_ = 6.23, respectively. Therefore, in the 1–8 pH range the mole fractions of the different forms (protonated, monoanion, dianion) change continuously with pH. The *k*_Cl2•-_ of fumaric acid shows the tendency protonated form<monoanion<dianion (1.2±1.0 × 10^5^, 2.0±0.3 × 10^6^ and 4.5±0.3 × 10^6^ M^-1^ s^-1^) in agreement with the electrophilic character of reaction. Neutral maleic acid has somewhat higher reactivity (1.7±0.2 × 10^6^ M^-1^ s^-1^) as the monoanion (1.25±0.1 × 10^6^ M^-1^ s^-1^). This is attributed to a prevalence of steric or polar effects for the monoanion. Acrylic acid, n-butyl acrylate, 3-sulfo propylmethacrylate, acrylamide, N-isopropyl acrylamide, and acrylonitrile (monomers used in polymerization reactions) have rate constants in the 10^6^–10^7^ M^-1^ s^-1^ range.

Muconic acid, a metabolite of benzene, is an important intermediate of chemical industry, sorbic acid is natural food preservative. These two compounds, due to the two conjugated double bonds in their structures react with rate constants in the 10^8^ M^-1^ s^-1^ range (Hasegawa and Neta 1978). 3-Hexenedioic acid (used e.g., in nylon production), reacts with *k*_Cl2•-_ in the same order of magnitude as the previous compounds, although it has only one double bond.

### Small organic molecules containing halogen atoms

Organic acids dissolve in water to form anions which can transfer an electron to oxidizing radicals (e.g., Cl_2_^•-^). The radical formed in the reaction undergoes decarboxylation (Maruthamuthu et al. 1995):

16 $$ \kern1em -2{\mathrm{Cl}}^{-}{\mathrm{CH}}_{3-\mathrm{n}}{\mathrm{X}}_{\mathrm{n}}{\mathrm{CO}\mathrm{O}}^{-}+{{\mathrm{Cl}}_2}^{\bullet -}---\to {\mathrm{CH}}_{3-\mathrm{n}}{\mathrm{X}}_{\mathrm{n}}{\mathrm{CO}\mathrm{O}}^{\bullet }---\to {\mathrm{CH}}_{3-\mathrm{n}}{{\mathrm{X}}_{\mathrm{n}}}^{\bullet }+{\mathrm{CO}}_2 $$

Chloroacetic acids are disinfection by-products in water. Mono-, di-, and trichloroacetic acids react with Cl_2_^•-^ with rate constants of 1.5 × 10^4^-6.5 × 10^4^ M^-1^ s^-1^ (Scheme 3S). The values of fluoroacetates, except monofluoroacetate, are lower than those of chloroacetates. 1,1,1-Trifluoro-2-iodoethane reacts with Cl_2_^•-^ in electron transfer, but it does not react with Br_2_^•-^ or I_2_^•-^ (Mohan et al. 1994). The *k*_Cl2•-_’s of trichloroethylene and diiodomethane are higher by two-three orders of magnitude than those of the previously mentioned compounds (Mohan and Moorthy 1990b; Mohan et al. 1994). Cl_2_^•-^ oxidizes CH_2_I_2_ to CH_2_I_2_^+^.

## Aromatic molecules

### Simple aromatic molecules

Benzene practically does not react with Cl_2_^•-^, *k*_Cl2•-_ ≤ 10^5^ M^-1^ s^-1^ (Alegre et al. 2000) (Table 2, Scheme 4S). Small values are suggested also for toluene, benzoic acid, chlorobenzene and benzonitrile (Hasegawa and Neta 1978; Mártire et al. 2001). In the latter two compounds the electron withdrawing group on the ring is expected to decrease the reactivity. Hasegawa and Neta attempted to find a correlation between the *k*_Cl2•-_ values and the Hammett parameters including in the investigations also compounds with electron donating groups (anisole, phenol, aniline). This attempt failed because two different mechanisms appear to be involved in the reactions with Cl_2_^•-^, i.e., addition to the aromatic ring and direct oxidation by electron transfer.

**Table 2:** Aromatic molecules

Compound, p*K*_a_	*k*_Cl2•-_, M^-1^ s^-1^	Method, pH	Literature
*Simple aromatic molecules*			
Benzene	<10^5^	FP, 4	Alegre et al. (2000)
Toluene	≤10^6^	FP, 4	Mártire et al. (2001)
γ-Cyclodextrine/Co_60_ complex	3.8 × 10^9^	Pr, ~1	Priyadarsini et al. (1994)
Chlorobenzene	≤10^6^	FP, 4	Mártire et al. (2001)
Iodobenzene	3.5 × 10^8^	Pr, 1.5	Mohan and Moorthy (1989)
Benzonitrile	<10^5^	Pr, 1-7	Hasegawa and Neta (1978)
*Anisoles*			
Anisole	1.62±0.09 × 10^8^	LFP, 7	Lei et al. (2019)
Thioanisole	4.8 × 10^9^	Pr, 1	Mohan and Mittal (1997)
2-(Phenylthio)ethanol	3.5 × 10^9^	Pr, 1	Gawandi et al. (1999a)
4-Nitroanisole	2.5±0.3 × 10^7^	LFP, 7	Lei et al. (2019)
1,3,5-Trimethoxybenzene (TMB)	2.87±0.26 × 10^9^	LFP, 7	Lei et al. (2019)
	2.61±0.17 × 10^9^	LFP, 7	
Atenolol, 9.5	9.81 × 10^6^	Comp., 5.8	Mangalgiri et al. (2019)
	4.11±0.24 × 10^8^	LFP, 7	Lei et al. (2019)
	4.0±0.4 × 10^8^	Comp., 7	Pan et al. (2019)
Metoprolol, 9.4	2.2±0.4 × 10^8^	LFP, 5.5–6	Jasper et al. (2016)
	5.07±0.38 × 10^8^	LFP, 7	Lei et al. (2019)
Propranolol. 9.5	1.9±0.1 × 10^9^	LFP, 5.5–6	Jasper et al. (2016)
	1.78±0.06 × 10^9^	LFP, 7	Lei et al. (2019)
Napropamide	8.3±0.8 × 10^8^	Pr, 2.0	Cornelius (1998)
Methylchlorophenoxyacetic acid (MCPA), 3.07	2.6±0.3 × 10^8^	Pr, 2.3	Cornelius (1998)
Triclosan, 7.9	2.48±0.14 × 10^8^	LFP, 7	Lei et al. (2019)
Gemfibrozil, 4.5	2.87±0.12 × 10^8^	LFP, 7	Lei et al. (2019)
Venlafaxine, 9.26	3.58±0.14 × 10^8^	LFP, 7	Lei et al. (2019)
*Anilines*			
Aniline, 4.8	1.2 × 10^7^	Pr, 1	Hasegawa and Neta (1978)
	6.79±0.45 × 10^8^	LFP, 7	Lei et al. (2019)
4-Toluidine, 5.0	9.47±0.52 × 10^8^	LFP, 7	Lei et al. (2019)
4-Chloroaniline, 3.98	5.23±0.23 × 10^8^	LFP, 7	Lei et al. (2019)
Acetanilide, 0.61	~2 × 10^7^	Pr, 7	Hasegawa and Neta (1978)
3-Aminophenol, 4.3, 9.83	3.6 × 10^8^	Pr, 1.0	Dwibedy et al. (2005)
4-Aminophenol, 5.4, 10.4	4 × 10^9^	Pr, 1.0	Dwibedy et al. (2005)
Acetaminophen, 9.5	<5 × 10^7^	LFP, 5.5–6	Jasper et al. (2016)
	4.32±0.07 × 10^8^	LFP, 7	Lei et al. (2019)
	4.4 × 10^8^	Complex	Wang et al. (2019)
2-Methylaminobenzoate, 2.12	4.0±0.3 × 10^8^	LFP, 3	Lanzafame et al. (2017)
Diclofenac, 4.2	1.15±0.05 × 10^9^	LFP, 7	Lei et al. (2019)
Indomethacin, 4.2	4.99±0.51 × 10^8^	LFP, 7	Lei et al. (2019)
Benzidine, 3.6, 5.7	7.0 × 10^7^	Pr, 2	Dey et al. (1994a)

The I-atom in iodobenzene has weak electron withdrawing properties: Mohan and Moorthy (1989) published much higher rate constant (3.5 × 10^8^ M^-1^ s^-1^) as the *k*_Cl2•-_ values of other simple aromatics. Iodobenzene reacts with Cl_2_^•-^ by one-electron oxidation:

17 $$ {{\mathrm{C}\mathrm{l}}_2}^{\bullet -}+{\mathrm{C}}_6{\mathrm{H}}_5\mathrm{I}\hbox{---} \to {\mathrm{C}}_6{\mathrm{H}}_5{\mathrm{I}}^{\bullet +}+2\ {\mathrm{C}\mathrm{l}}^{-} $$

Similar oxidation was not observed in reaction with Br_2_^•-^: the reduction potentials of Cl_2_^•-^ and Br_2_^•-^ are published to be 2.1 and 1.60, respectively, *vs*. NHE (Table 1S). The reduction potential of C_6_H_5_I^•+^/C_6_H_5_I couple is suggested as >2.0 V.

The reaction of water insoluble Co_60_ (Buckminsterfullerene) was investigated as a soluble γ-cyclodextrine/Co_60_ complex, the fullerene is suggested to be enclosed by two γ-cyclodextrine molecules. The reaction produces a transient species that was assigned as ^•^Co_60_Cl radical adduct (Priyadarsini et al. 1994), rate constant: 3.8 × 10^9^ M^-1^ s^-1^.

### Anisoles

The *k*_Cl2•-_ of anisole and, in general of aromatic molecules with side chains connected to the ring through an O-atom (Scheme 5S) are higher than those of the simple aromatic molecules. The *k*_Cl2•-_ of anisole (1.62±0.09 × 10^8^ M^-1^ s^-1^) and 4-nitroanisole (2.5±0.3 × 10^7^ M^-1^ s^-1^) nicely exemplify the effect of electron density on the ring. The *k*_Cl2•-_ of 4-nitroanisole with electron withdrawing –NO_2_ group is one order of magnitude smaller than that of the molecule without this substituent. The three methoxy groups in 1,3,5-trimethoxybenzene by enhancing the electron density on the ring increase the rate constant of electrophile reactions to a high value, *k*_Cl2•-_ = 2.87±0.26 × 10^9^ M^-1^ s^-1^ (Lei et al. 2019). As it will be discussed later Cl_2_^•-^ has high reactivity with sulfur atoms in lower oxidation state in organic molecules. Thioanisole and 2-(phenylthio) ethanol react with Cl_2_^•-^ with rate constants of 4.8 × 10^9^ and 3.5 × 10^9^ M^-1^ s^-1^, respectively (Mohan and Mittal 1997; Gawandi et al. 1999a).

The blood pressure regulators (β-blockers) atenolol, metoprolol and propranolol have common structure of R-O-CH_2_-CH(OH)-CH_2_-NH-CH(CH_3_)_2_ with optical centers at the OH group on the alkyl chain (R is aromatic). At neutral pH they have positive charge on the N-atom (p*K*_a_ ≈ 9.5). The rate constants of reactions with Cl_2_^•-^ are in the 10^8^–10^9^ M^-1^ s^-1^ range (Jasper et al. 2016; Lei et al. 2019; Pan et al. 2019). These values in reaction with SO_4_^•-^ are around 1 × 10^10^ M^-1^ s^-1^, while those of reactions with CO_3_^•-^ are in the 2 × 10^6^–5 ×10^7^ M^-1^ s^-1^ range (Wojnárovits and Takács 2019; Wojnárovits et al. 2020). The rate constants increasing in the CO_3_^•-^<Cl_2_^•-^<SO_4_^•-^ order are in agreement with the increasing reduction potential of oxidant (Table 1S). The similar rate constants of the three molecules in all three one-electron oxidations reflect reactions occurring on the same center, mainly on the aromatic rings.

Napropamide (Nap, herbicide), reacts with Cl_2_^•-^ and Br_2_^•-^, and does not react with (SCN)_2_^•-^ (*E*^0^(SCN)_2_^•-^/2(SCN)^-^ = 1.30 V *vs*. NHE) and N_3_^•^ (*E*^0^(N_3_^•^/N_3_^-^) = 1.33 V *vs*. NHE). Therefore, the reduction potential of the Nap^•+^/Nap couple must lie between 1.63 and 1.33 V *vs* NHE. The reduction potential of the phenoxy type herbicide MCPA (2-methyl-4-chlorophenoxyacetic acid) is expected to be between 2.1 and 1.66 V *vs*. NHE. It reacts with Cl_2_^•-^ (2.6±0.3 × 10^8^ M^-1^ s^-1^), but does not react with Br_2_^•-^ (*E*^0^(Br_2_^•-^/2Br^-^) = 1.63 V *vs*. NHE) (Cornelius 1998). Triclosan is used to prevent or reduce bacterial contamination. Lei et al. (2019) published a relatively high rate constant for the partly ionized molecule: 2.48±0.14 × 10^8^ M^-1^ s^-1^.

Gemfibrozil is used to treat abnormal blood lipid levels. It reacts with *k*_Cl2•-_ of 2.87±0.12 × 10^8^ M^-1^ s^-1^ (Lei et al. 2019) that is slightly smaller than the rate constant of reaction with SO_4_^•-^ (7.13±0.78 × 10^8^ M^-1^ s^-1^, Lian et al. 2017), but much higher than the rate constant of CO_3_^•-^ reaction (4.1±3.2 × 10^6^ M^-1^ s^-1^, Wols et al. 2014). Venlafaxine is used to treat depressive disorder. Its *k*_Cl2•-_ is published as 3.58±0.14 × 10^8^ M^-1^ s^-1^ (Lei et al. 2019). This value is between the rate constants measured in CO_3_^•-^ and SO_4_^•-^ reactions: 4.89±2.31 × 10^6^ and 3.53±0.05 × 10^9^ M^-1^ s^-1^, respectively (Lian et al. 2017). The *k*_Cl2•-_ values of gemfibrizil and venlafaxine are close to that of anisole, the Cl_2_^•-^ attack is suggested on the benzene ring.

### Anilines

Aniline cation reacts in direct oxidation with *k*_Cl2•-_ = 1.2 × 10^7^ M^-1^ s^-1^ (Hasegawa and Neta 1978). An OH group added to aniline in *meta*-position (Scheme 6S) in 3-amino-phenol (3-APH^+^) increases *k*_Cl2•-_ to 3.6 × 10^8^ M^-1^ s^-1^ (Dwibedy et al. 2005): in the reaction phenoxyl radical forms just as in reactions of other one-electron oxidants. There is no reaction between Br_2_^•-^ and 3-APH^+^ indicating a reduction potential of the 3-AP^•^,H^+^/3-APH^+^ couple between 2.1 and 1.63 V *vs*. NHE.

The rate constant of reaction with the neutral aniline molecule is much higher, 6.79±0.45 × 10^8^ M^-1^ s^-1^ (Lei et al. 2019) than that with the cation. The *k*_Cl2•-_ of 4-chloroaniline, 5.23±0.23 × 10^8^ M^-1^ s^-1^ is nearly as high as that of aniline. The electron withdrawing Cl-atom does not decrease the reactivity. The methyl group in 4-toluidine increases *k*_Cl2•-_ to 9.47±0.52 × 10^8^ M^-1^ s^-1^. The *k*_Cl2•-_ of acetanilide is an order of magnitude smaller (~2 × 10^7^ M^-1^ s^-1^, Hasegawa and Neta 1978) than that of neutral aniline. The acetyl group attached to amino group decreases the reactivity also in reactions with other radicals, e.g., CO_3_^•-^ (Wojnárovits et al. 2020). Moderately high value was measured for methyl 2-methyl aminobenzoate (methyl anthranilate, flavouring agent), 4.0±0.3 × 10^8^ M^-1^ s^-1^ (Lanzafame et al. 2017).

Acetaminophen (paracetamol, medicine used to relief pain) is an aminophenol derivative. These derivatives (e.g., 2-, 3-, and 4-aminophenols, amodiaquine type antimalarial drugs) represent special cases of one-electron oxidation. In these reactions, phenoxyl radical forms in which a part of spin density is concentrated on the N-atom. This radical is also called the aminophenoxy radical (Bisby and Tabassum 1988; Bisby 1990; Szabó et al. 2012). For the acetaminophen + Cl_2_^•-^ reaction highly different values were published by Jasper et al. (2016) and Lei et al. (2019): <5 × 10^7^ and 4.32±0.07 × 10^8^ M^-1^ s^-1^, respectively. Wang et al. (2019) in a complex kinetic system estimated *k*_Cl2•-_ ≈ 4.4 × 10^8^ M^-1^ s^-1^. Dwibedy et al. (2005) published unusually high rate constant (4 × 10^9^ M^-1^ s^-1^) for Cl_2_^•-^ reaction with 4-aminophenol cation.

Diclofenac is a frequently used nonsteroidal antiinflammatory medicine. In the molecule aromatic rings are connected through an –NH– bridge. One-electron oxidants attack this bridge by removing an electron (Wojnárovits and Takács 2019). Due to this possibility in Cl_2_^•-^ reaction high rate constant was measured: 1.15±0.05 × 10^9^ M^-1^ s^-1^ (Lei et al. 2019). In indomethacin (also used as nonsteroidal antiinflammatory drug) an aromatic pyrrole ring is fused to a benzene ring. The radical attack is expected on this fused part of the molecule, *k*_Cl2•-_ is moderately high, 4.99±0.51 × 10^8^ M^-1^ s^-1^ (Lei et al. 2019).

Benzidine (1,1'-biphenyl-4,4’-diamine) and its derivatives are used as intermediates in dye production. They are carcinogenic and mutagenic (McClelland et al. 2000). *k*_Cl2•-_ belonging to the singly ionized form (7 × 10^7^ M^-1^ s^-1^, pH 7, Dey et al. 1994a) is between the values measured for protonated and nonprotonated aniline.

## Phenols and benzoic acids

The rate constant of Cl_2_^•-^ reaction with phenol has been reported in four laboratories (Table 3, Scheme 7S), the values are close to each-other with average of 3.8±1.0 × 10^8^ M^-1^ s^-1^ (Willson 1973; Hasegawa and Neta 1978; Alfassi et al. 1990; Lei et al. 2019). In the reaction chlorinated phenols were observed among the final products; these highly poisonous compounds underline the importance of studying Cl_2_^•-^ reactions. As intermediate phenoxyl radical was identified. Alfassi et al. (1990) investigating the temperature dependence of *k*_Cl2•-_ found an increasing tendency as the temperature increased with Arrhenius parameters of *E*_a_ = 7.1±0.6 kJ mol^-1^ and Log *A* = 9.8. Estrone, a steroid hormone with phenol part, reacts with similar *k*_Cl2•-_ (3.66±0.24 × 10^6^ M^-1^ s^-1^, Lei et al. 2019) as phenol (3.8±1.0 × 10^6^ M^-1^ s^-1^). The radical is assumed to attack the aromatic ring.

**Table 3:** Phenols and benzoic acids.

Compound, p*K*_a_	*k*_Cl2•-_, M^-1^ s^-1^	Method, pH	Literature
Phenol, 10.0	5 × 10^8^	Pr, 1–2	Willson (1973)
	2.5 × 10^8^	Pr, 1	Hasegawa and Neta (1978)
	3.2 × 10^8^	Pr, 2.5	Alfassi et al. (1990)
	2.2±0.1 × 10^8^	LFP, 7	Lei et al. (2019)
4-Methoxyphenol, 10.05	1.1±0.1 × 10^9^	Pr, 1	Hasegawa and Neta (1978)
4-Cyanophenol, 7.97	4.0±0.4 × 10^7^	Pr, 1	Hasegawa and Neta (1978)
Dichlorophen, 7.6	4.2 × 10^8^	Pr, 2.0	Cornelius (1998)
Ioxynil, 4.1	1.25±0.13 × 10^9^	Pr, 2.0	Cornelius (1998)
Estrone, 10.7	3.66±0.24 × 10^8^	LFP, 7	Lei et al. (2019)
Hydroquinone, 9.9	1.4±0.2 × 10^9^	Pr, 1	Hasegawa and Neta (1978)
	1 × 10^9^	Pr	Willson (1973)
Catechol, 9.3	5.66±0.41 × 10^8^	LFP, 7	Lei et al. (2019)
4-Methylcatecol, 9.9	1.18±0.03 × 10^9^	LFP, 7	Lei et al. (2019)
Dopamine, 10.0	~2 × 10^9^	Pr, 1.5	Maity et al. (1994a)
4-Hydroxybenzyl alcohol, 9.8	2.3 × 10^8^	Pr, 1	Dhiman and Naik (2010)
4-Hydroxycinnamic acid, 4.1	2.9 × 10^8^	Pr, 2.9	Bobrowski (1984)
Bisphenol A, 9.6	5.82±0.62 × 10^8^	LFP, 7	Lei et al. (2019)
	4.6 × 10^6^	Pr	Fang et al. (2020)
4-Methylthiophenol, 6.8	4.7 × 10^9^	Pr, 0	Dey et al. (1999)
4,4’-Thiodiphenol, 9.2	6.9 × 10^9^	Pr, 1	Mohan and Mittal (1999)
Benzoic acid, 4.2	2 × 10^6^	Pr, 7	Hasegawa and Neta (1978)
	≤10^6^	FP, 4	Mártire et al. (2001)
	2±0.3 × 10^6^	LFP, 7	Lei et al. (2019)
4-Aminobenzoic acid, 2.35, 4.98	2.2±0.2 × 10^7^	Pr, 1	Hasegawa and Neta (1978)
	1.1±0.1 × 10^9^	Pr, 7	
4-Methoxybenzoic acid, 4.47	2.0±0.2 × 10^6^	Pr, 7	Hasegawa and Neta (1978)
3-Methylbenzoic acid, 4.27	6.81 × 10^4^	Complex, 7.2	Zhou et al. (2019)
4-Methylbenzoic acid, 4.37	5±1 × 10^6^	Pr, 7	Hasegawa and Neta (1978)
4-Fluorobenzoic acid, 4.14	5.20 × 10^4^	Complex, 7.2	Zhou et al. (2019)
2-Chlorobenzoic acid, 2.9	3.0 × 10^4^	Complex, 7.2	Zhou et al. (2019)
4-Chlorobenzoic acid, 4.07	3.0±0.6 × 10^6^	Pr, 7	Hasegawa and Neta (1978)
4-Bromobenzoic acid, 4.0	7±1 × 10^6^	Pr, 7	Hasegawa and Neta (1978)
2-Iodobenzoic acid, 2.86	2.0 × 10^4^	Complex, 7.2	Zhou et al. (2019)
4-Cyanobenzoic acid, 3.6	5±1 × 10^6^	Pr, 7	Hasegawa and Neta (1978)
3-Cyanobenzoic acid, 3.6	1.89 × 10^4^	Complex, 7.2	Zhou et al. (2019)
3-Nitrobenzoic acid, 3.4	1.08 × 10^4^	Complex, 7.2	Zhou et al. (2019)
4-Hydroxybenzoic acid, 4.5	1.1±0.1 × 10^8^	Pr, 1	Hasegawa and Neta (1978)
	1.3±0.1 × 10^8^	Pr, 3.1	
	2.8±0.2 × 10^8^	Pr, 7	
	1.5±0.1 × 10^9^	Pr, 9.5	
4-Phenoxybenzoic acid, 4.6	1.5±0.2 × 10^8^	Pr, 7	Hasegawa and Neta (1978)
Salicylic acid, 3.0	1.1 × 10^8^	Pr, 1	Hasegawa and Neta (1978)
	2.10±0.22 × 10^8^	LFP, 7	Lei et al. (2019)
5-Aminosalicylic acid, 2.7, 5.8	1.42±0.03 × 10^9^	LFP, 7	Lei et al. (2019)
Acetylsalicylic acid, 3.4	6.8±1.4 × 10^7^	LFP, 7	Lei et al. (2019)
Gallic acid, 4.4, 8.2, 10.7, 13.1	1.9 × 10^9^	Pr, 0	Dwibedy et al. (1999)
	7.53±0.43 × 10^8^	LFP, 7	Lei et al. (2019)
Propyl gallate, 8.1	2 × 10^9^	Pr, 1–2	Willson (1973)
Methyl paraben, 8.2	1.61±0.06 × 10^8^	LFP, 7	Lei et al. (2019)
Protocatechuic acid, 4.4, 8.7	5.90±0.31 × 10^8^	LFP, 7	Lei et al. (2019)
Terephthalic acid, 3.51, 4.82	6±1 × 10^6^	Pr, 7	Hasegawa and Neta (1978)
Dimethyl phthalate, 3.4	1.4±0.3 × 10^7^	LFP, 7	Lei et al. (2020)
Diethyl phthalate, 7.8	1.1±0.2 × 10^7^	LFP, 7	Lei et al. (2020)
Dibutyl phthalate, 3.8	1.1±0.2 × 10^7^	LFP, 7	Lei et al. (2020)
Ibuprofen, 4.9	<5 × 10^6^	LFP, 7	Lei et al. (2019)
Naproxen, 4.2	6.57±0.43 × 10^8^	LFP, 7	Lei et al. (2019)
4-(Methylthio)benzoic acid, 3.48	2.51 × 10^9^	Pr, 1	Gawandi et al. (2003)

Hydroquinone is more reactive in reaction with Cl_2_^•-^ (1.4±0.2 × 10^9^ M^-1^ s^-1^, Hasegawa and Neta 1978) than catechol (5.66±0.41 × 10^8^ M^-1^ s^-1^, Lei et al. 2019). In hydroquinone reaction, similarly to phenol, also phenoxyl radical intermediate was observed. The rate constants of 4-methylcatechol and dopamine (3,4-dihydroxyphenethylamine) (1.18±0.03 × 10^9^ and ~2 × 10^9^ M^-1^ s^-1^, respectively, Maity et al. 1994a; Lei et al. 2019), with electron donating group on the ring, are as high as that of hydroquinone. In cyanophenol electron withdrawing CN decreases *k*_Cl2•-_ as compared to that of phenol by an order of magnitude (Hasegawa and Neta 1978).

Dichlorophen (fungicide, germicide, and antimicrobial agent) and ioxynil (herbicide) react with *k*_Cl2•-_ of 4.2 × 10^8^ and 1.25±0.13 × 10^9^ M^-1^ s^-1^, respectively. They react also with SO_4_^•-^, Br_2_^•-^ and N_3_^•^, but do not react with I_2_^•-^ (*E*^0^(I_2_^•-^/2I^-^) = 1.04 V *vs*. NHE). Therefore, their reduction potentials are between 1.33 and 1.04 V *vs*. NHE (Cornelius 1998). The bimolecular rate constant decreases with the oxidizing power of the attacking radical.

In 4-hydroxybenzyl alcohol a CH_2_-OH, in 4-hydroxycinnamic acid a CH=CH-COOH group in *para*-position on the ring practically does not influence the rate constant. The values, 2.3 × 10^8^ and 2.9 × 10^8^ M^-1^ s^-1^, respectively (Bobrowski 1984; Dhiman and Naik 2010), are close to the *k*_Cl2•-_ of phenol. The 4-hydroxybenzyl alcohol + Cl_2_^•-^ reaction gives phenoxyl radical. Build-up of this radical was used in rate constant determination (Dhiman and Naik 2010). The mechanisms of reactions were suggested as simple electron transfer. The rate constants of the sulfur compounds, 4-methylthiophenol and 4,4’-thiodiphenol, 4.7 × 10^9^ and 6.9 × 10^9^ M^-1^ s^-1^, respectively, are much higher than those of phenols (Dey et al. 1999; Mohan and Mittal 1999).

Benzoic acid at the pH in the measurements of Mártire et al. (2001) was mainly in the neutral, while in the investigations of Hasegawa and Neta (1978) and Lei et al. (2019) in the anionic form (Scheme 8S). The *k*_Cl2•-_ of the anionic form (2 × 10^6^ M^-1^ s^-1^) is higher than that of the neutral molecule (<10^6^ M^-1^ s^-1^). Zhou et al. (2019) using a complex reaction system and a multicomponent fitting procedure for 3-methyl-, 4-fluoro-, 2-chloro-, 2-iodo-, 3-cyano-, and 3-nitrobenzoate (pH 7.2) estimated *k*_Cl2•-_ values in the 10^4^ M^-1^ s^-1^ range: log *k*_Cl2•-_ linearly correlated with the Hammett substituents constants with slope −0.96. The high negative slope reflects considerable selectivity.

The rate constants of 4-hydroxybenzoic and 4-phenoxybenzoic acids are two orders magnitude higher than those of the previously mentioned benzoic acid derivatives due to the increased electron density on the rings. As the measurements of Hasegawa and Neta (1978) with 4-hydroxybenzoic acid show that *k*_Cl2•-_ is increasing with increasing pH, the highest value was measured at pH 9.5, 1.5±0.1 × 10^9^ M^-1^ s^-1^.

The neutral form of salicylic acid has also smaller *k*_Cl2•-_ as the ionic species (1.1 × 10^8^ and 2.1±0.3 × 10^8^ M^-1^ s^-1^, respectively, Hasegawa and Neta 1978; Lei et al. 2019), similarly to benzoic acid. Both values are higher than the *k*_Cl2•-_ of benzoic acid due to the OH group in *ortho*-position in salicylic acid. The *k*_Cl2•-_ of acetylsalicylic acid (aspirin, medication used to treat inflammation) is in the same order of magnitude as that of salicylic acid. The *k*_Cl2•-_ of 5-aminosalicylic acid (mesalazine, antiinflammatory drug) is high (1.42±0.03 × 10^9^ M^-1^ s^-1^), here the amino group increases the electron density on the ring. *k*_Cl2•-_ increases to 1.9 × 10^9^ M^-1^ s^-1^ in gallic acid (neutral molecule) with three OH groups in *meta* and *para* positions (Dwibedy et al. 1999). The *k*_Cl2•-_ of propyl gallate (propyl 3,4,5-trihydroxybenzoate, 2 × 10^9^ M^-1^ s^-1^) is similar to that of gallic acid (Willson 1973). The *k*_Cl2•-_ values of protocatechuic acid (3,4-dihydroxybenzoic acid, antiviral agent) and gallic acid, with dissociated carboxylic groups (5.9±0.31 × 10^8^ and 7.53±0.43 × 10^8^ M^-1^ s^-1^, respectively, Lei et al. 2019) are similar.

Parabens, *p*-hydroxybenzoic acid derivatives, are widely used as preservatives in cosmetic and pharmaceutical products because of their bactericidal and fungicidal properties. For the reaction of methyl paraben, Lei et al. (2019) measured a *k*_Cl2•-_ of 1.61±0.06 × 10^8^ M^-1^ s^-1^. Terephthalic acid (1,4-benzenedicarboxylic acid) is mainly used as raw material for polyester production. It reacts with a *k*_Cl2•-_ that is similar to that of benzoic acid (2 × 10^6^ M^-1^ s^-1^, Hasegawa and Neta 1978). Phthalate esters (1,2-benzenedicarboxylic acid esters) are widely used as plasticizers in the plastic industry. Some of the phthalate esters have endocrine-disrupting properties which restrict their applications. Lei et al. (2019) for dimethyl-, diethyl-, and dibutyl phthalate published *k*_Cl2•-_ values around 1.2 × 10^7^ M^-1^ s^-1^.

Ibuprofen and naproxen are used as nonsteroidal antiinflammatory drugs. The former has central benzene the latter naphthalene unit. At the pH of *k*_Cl2•-_ determination (pH 7) in both molecules the carboxyl group is ionized. Lei et al. (2019) suggest a rate constant of ibuprofen reaction smaller than 5 × 10^6^ M^-1^ s^-1^, for naproxen they measured 6.77±0.43 × 10^8^ M^-1^ s^-1^. It is easier to ionize the naphthalene ring than the benzene ring. In reaction with SO_4_^•-^, the rate constants measured for the two compounds (1.3 × 10^9^–3 × 10^9^ and 5.6 × 10^9^–(7–8) × 10^9^ M^-1^ s^-1^, respectively) are close to each-other (Paul et al. 2014; Kwon et al. 2015; Yang et al. 2017; Gao et al. 2017). This is due to the closeness of the diffusion controlled limit for the SO_4_^•-^ reaction (6.5 × 10^9^ M^-1^ s^-1^, Wojnárovits and Takács 2019), which supresses the rate constant range. In CO_3_^•-^ reaction the rate constant of ibuprofen is also smaller than that of naproxen (~1 × 10^6^ and 5.6±1.1 × 10^7^ M^-1^ s^-1^, respectively) (Wols et al. 2014; Lian et al. 2017).

The rate constant of 4-(methylthio)benzoic acid (2.51 × 10^9^ M^-1^ s^-1^) is much higher than those of the other benzoic acid derivatives (Gawandi et al. 2003).

## Sulfur compounds

The reactions of sulfur compounds (organic sulfides, sulfoxides) represent special cases in radical reactions (Table 4, Scheme 9S). One-electron oxidants easily oxidize the S-atom in lower oxidation state to a higher oxidation state sulfur. Sulfur radical cations show high tendency to be stabilized through three-electron bonded S**∴**X (X=O, S, N, Cl, Br, I) species. The *k*_Cl2•-_ values of organic sulfide (R_1_R_2_S) reactions are in the 10^8–^10^9^ M^-1^ s^-1^ range (Bonifacic and Asmus 1980; Mohan 1990; Mohan and Moorthy 1990a; Mohan and Mittal 1991, 1992; Maity et al. 1994b). In the experiments, Cl-adduct, R_1_R_2_S**∴**Cl, intermediates were observed. These species are characterized by a sulfur-chlorine three-electron bond with two σ-bonding and one σ*-antibonding electron. An equilibrium exists between Cl_2_^•-^ and R_1_R_2_S**∴**Cl (18). Three-electron bonded complex (dimer radical cation) also forms in the R_1_R_2_S**∴**Cl + R_1_R_2_S Reaction (19).

**Table 4:** Sulfur compounds

Compound, p*K*_a_	*k*_Cl2•-_, M^-1^ s^-1^	Method, pH	Literature
Dimethyl sulphide	4.7 × 10^8^	Pr, <3	Bonifacic and Asmus (1980)
Diethyl sulphide	1.8 × 10^8^	Pr, <3	Bonifacic and Asmus (1980)
Methionine, 9.21, 9.28	3.9 × 10^9^	Pr, 1	Hiller and Asmus (1981)
Methionine anhydride	5.8±1.0 × 10^9^	Pr, 1	Holcman et al. (1991)
Methionine methyl ester, 9.2	6.1 × 10^9^	Pr, 1	Shirdhonkar et al. (2006)
Thiodiglycolic acid	4.9 × 10^8^	Pr, 1.5	Mohan and Moorthy (1990a)
2,2'-Thiodiethanol, 3.32, 4.29	1.1 × 10^9^	Pr, 1.5	Mohan and Mittal (1991)
Dimethyl 3,3’-thio-dipropionic acid, 4.0, 5.0	1.2 × 10^9^	Pr, 1.5	Mohan and Mittal (1992)
3,3’-Thiodipropionol	2.6 × 10^9^	Pr, 1.5	Mohan and Mittal (1992)
Dimethyl 2,2’-thiodiethanoate	1.9 × 10^9^	Pr, 1.5	Maity et al. (1994b)
Methylthioacetic acid, 3.4	2.8 × 10^9^	Pr, 1	Gawandi et al. (2000)
2-(Methylthio)ethanol	2.1 × 10^9^	Pr, 1	Gawandi et al. (1999b)
3-(Methylthio)propanol	2.2 × 10^9^	Pr, 1	Gawandi et al. (1999b)
4-(Methylthio)butanol	1.9 × 10^9^	Pr, 1	Gawandi et al. (1999b)
3,3’-Thiodipropionamide	7.1 × 10^9^	Pr, 1	Gawandi et al. (1999b)
(4-Methylthiophenyl) methanol	3.1 × 10^9^	Pr, 1	Mohan and Mittal (2001)
Dithiothreitol, 9.2, 10.1.	3.0±0.3 × 10^9^	Pr, 2.0	Redpath (1973)
Cimetidine, 6.8	3.0±0.2 × 10^9^	LFP, 5.5-6	Jasper et al. (2016)
	2.78±0.16 × 10^9^	LFP, 7	Lei et al. (2019)
Famotidine, 1.8, 6.8	1.65±0.04 × 10^9^	LFP, 7	Lei et al. (2019)
Ranitidine, 2.7, 8.2	4.5±0.5 × 10^9^	LFP, 5.5-6	Jasper et al. (2016)
Methidation	1.3±0.4 × 10^8^	FP, 5.5	Caregnato et al. (2013)
Dimethoate	1.1±0.4 × 10^8^	FP, 5.5	Caregnato et al. (2013)
Phenyl trifluoromethyl sulfide (PTS)	≤1.5 × 10^7^	Pr, 1	Shirdhonkar et al. (2008)
Dimethyl sulfoxide	1.2 × 10^7^	Pr, 1.7	Kishore and Asmus (1991)
	1.6±0.8 × 10^7^	LFP, 5-6	Zhu et al. (2005)
Diethyl sulfoxide	2.7 × 10^7^	Pr, 1.7	Kishore and Asmus (1991)
Dipropyl sulfoxide	3.9 × 10^7^	Pr, 1.7	Kishore and Asmus (1991)
Dimethyl sulfone	8.2±5.5 × 10^3^	LFP, 5-6	Zhu et al. (2005)
Methanesulfonate	3.9±0.7 × 10^3^	LFP, 5-6	Zhu et al. (2005)
Methanesulfinate	8.0±1.0 × 10^8^	LFP, 5-6	Zhu et al. (2005)
Thiazole, 2.5	3.9±0.2 × 10^8^	LFP, 7	Lei et al. (2019)
Diethylthiourea	4.0 × 10^9^	Pr, 0	Dey et al. (1994c)
Phenylthiourea, 13.9	4.0 × 10^9^	Pr, 2.0	Dey et al. (1994b)
n-Allylthiourea, 13.6	4.6 × 10^9^	Pr, 1	Naik and Mukherjee (2006)
Selenothiourea,8.6	3.6 × 10^9^	Pr, 1	Mishra et al. (2004)
2-Mercapto-benzimidazole, 10.9	8.8 × 10^9^	Pr, 0	Dey et al. (1995)
Thioacetamide	2.5 × 10^9^	Pr, 0	Kishore et al. (1998)
Dithio-oxamide	4 × 10^9^	Pr	Dey et al. (2000)
Thionicotinamide, 3.25	1.3 × 10^9^	Pr, 1	Dey et al. (2003)
2,5-Dimer-captothiadiazole, 4.1, 9.4	7.0 × 10^8^	Pr. 0	Kishore et al. (1998)

18 $$ {{\mathrm{Cl}}_2}^{\bullet -}+{\mathrm{R}}_1{\mathrm{R}}_2\mathrm{S}\kern0.5em \rightleftarrows {\mathrm{R}}_1{\mathrm{R}}_2\mathrm{S}\therefore \mathrm{Cl}+{\mathrm{Cl}}^{-} $$

19 $$ {\mathrm{R}}_1{\mathrm{R}}_2\mathrm{S}\therefore \mathrm{Cl}+{\mathrm{R}}_1{\mathrm{R}}_2\mathrm{S}\rightleftarrows {\left({R}_1{\mathrm{R}}_2\mathrm{S}\therefore {\mathrm{SR}}_1{\mathrm{R}}_2\right)}^{+}+{\mathrm{Cl}}^{-} $$

Dithiothreitol (DTT) is a small-molecule redox reagent with a very low reduction potential of 0.33 V *vs*. NHE at pH 7 (Redpath 1973). It is often used in racemic form in the acidic pH range. DTT reacts with Cl_2_^•-^ with a rate constant of 3.0±0.3 × 10^9^ M^-1^ s^-1^.

Cimitidine, famotidine and ranitidine are used to control stomach acid overproduction. All contain sulfur atom in the alkyl chain. Based on analogous reactions, this S bridge is attacked in one-electron oxidation reaction giving explanation for the unusually high rate constants: 1.65 × 10^9^–4.5 × 10^9^ M^-1^ s^-1^ (Jasper et al. 2016; Lei et al. 2019). The use of methidation and dimethoate organophosphate insecticides are banned in several countries. In these molecules, the S-atom is connected to a thiophoshate group. The *k*_Cl2•-_ values were determined as 1.3±0.4 × 10^8^ and 1.1±0.4 × 10^8^ M^−1^ s^−1^, respectively. A mechanism involving charge transfer from the sulfide groups is proposed and supported by the identified intermediates and reaction products (Caregnato et al. 2013). In the reaction P(OCH_3_)SCl fragment forms. In phenyl trifluoromethyl sulfide (PTS) the CF_3_ group decreases the reactivity (Shirdhonkar et al. 2008).

Dimethyl sulfide oxidation in marine atmosphere may play an important role in modifying the global climate since several of its free radical induced oxidation products are water soluble (Zhu et al. 2005) contributing to atmospheric aerosols formation. The less oxidized sulfur compounds, dimethyl-, diethyl-, and di-n-propyl sulfoxides react with moderate *k*_Cl2•-_ of 1.2 × 10^7^–3.9 × 10^7^ M^-1^ s^-1^ (Kishore and Asmus 1991; Zhu et al. 2005). In reactions with alkylsulfoxides also three-electron bonded Cl-adducts were observed as intermediates (R_1_R_2_S(O)**∴**Cl, Scheme 2). These intermediates may form in one step (20) or two steps (21) reactions.

20 $$ {\mathrm{R}}_1{\mathrm{R}}_2\mathrm{S}\mathrm{O}+{{\mathrm{Cl}}_2}^{\bullet -}\hbox{---} \to \kern0.5em {\mathrm{R}}_1{\mathrm{R}}_2\mathrm{S}\left(\mathrm{O}\right)\therefore \mathrm{Cl}+{\mathrm{Cl}}^{-} $$

$$ \kern5em -{\mathrm{Cl}}^{-} $$

21 $$ {\mathrm{R}}_1{\mathrm{R}}_2\mathrm{S}\mathrm{O}+{{\mathrm{Cl}}_2}^{\bullet -}\hbox{---} \to {\mathrm{R}}_1{\mathrm{R}}_2{\mathrm{SO}}^{+}+{\mathrm{Cl}}^{-}\hbox{---} \to {\mathrm{R}}_1{\mathrm{R}}_2\mathrm{S}\left(\mathrm{O}\right)\therefore \mathrm{Cl} $$

The rate constants of reactions with the higher oxidized sulfur compounds, dimethyl sulfone and methanesulfonate are very small, they are 8.2±5.5 × 10^3^ and 3.9±0.7 × 10^3^ M^-1^ s^-1^, respectively (Zhu et al. 2005). However, a higher *k*_Cl2•-_ value, 8.0±1.0 × 10^8^ M^-1^ s^-1^, was published for methanesulfinate. As the authors mentioned, in their experiments the reactions of Cl^•^ might disturbe the determination of the rate constant.

Thiazole is the structural unit of several drugs, e.g., sulfathiazole, it reacts with moderately high rate constant of 3.9±0.2 × 10^8^ M^-1^ s^-1^ (Lei et al. 2019). Dey et al. (1994b, c) published high *k*_Cl2•-_ for the reaction of phenylthiourea and diethythiourea: 4.0 × 10^9^ M^-1^ s^-1^. High value was also found for n-allylthiourea (4.6 × 10^9^ M^-1^ s^-1^, Naik and Mukherjee 2006). Electron transfer, followed by deprotonation of the intermediate radical cation is suggested as a possible mechanism. It is proposed that an intramolecular 3-electron bond is formed between sulfur and nitrogen after the deprotonation. Such sulfur–nitrogen three-electron bond was also reported, e.g., in the radical reactions of methionine (Asmus 1990). A similar mechanism is suggested for Cl_2_^•-^ reaction with 2-mercaptobenzimidazole, *k*_Cl2•-_ is at the diffusion controlled limit: 8.8 × 10^9^ M^-1^ s^-1^ (Dey et al. 1995). High value was also published for selenourea (Mishra et al. 2004). The radical chemistry of thioacetamide is similar to that of thiourea, rather than that of acetamide (2.5 × 10^9^ M^-1^ s^-1^, Kishore et al. 1998).

## Antibiotics and model compounds

### β-Lactams

In these antibiotics the bicyclic system includes a thioether moiety, an especially susceptible part of the molecules in radical reactions (Szabó et al. 2016). 6-Aminopenicillanic acid and 4-hydroxy-D-phenyl glycine (Table 5, Scheme 10S) are regarded as model compounds of the more complex β-lactam antibiotics, e.g., amoxicillin (Song et al. 2008; Rickman and Mezyk 2010; Szabó et al. 2016). The rate constant of Cl_2_^•-^ reaction, measured for 6-aminopenicillanic acid at pH 2 is an order of magnitude higher (1.3 × 10^9^ M^-1^ s^-1^) than that determined for 4-hydroxy-D-phenyl glycine (1.8 × 10^8^ M^-1^ s^-1^). The attack on the former compound is suggested to take place on the sulfur atom giving rise to formation of three-electron bonded complexes. The reaction with 4-hydroxy-D-phenyl glycine is suggested to proceed by one-electron oxidation at the ring yielding a radical cation: the intermediate by deprotonation rearranges to phenoxyl radical (Szabó et al. 2016). It is evident that at pH 2 the reaction with amoxicillin occurs principally with the β-lactam part of the molecule (1.6 × 10^9^ M^-1^ s^-1^). Lei et al. (2019) measured much smaller value for amoxicillin reaction at pH 7 (4.20±0.11 × 10^8^ M^-1^ s^-1^).

**Table 5:** Antibiotics and model compounds

Compound, p*K*_a_	*k*_Cl2•-_, M^-1^ s^-1^	Method, pH	Literature
*β-lactams*			
6-Aminopenicillanic acid, 3.4	1.3 × 10^9^	Pr, 2	Szabó et al. (2016)
	3.27±0.31 × 10^8^	LFP, 7	Lei et al. (2019)
4-hydroxy-D-phenyl glycine, 2.15	2.8 × 10^8^	Pr, 2	Szabó et al. (2016)
Amoxicillin, 2.6, 7.2	1.6 × 10^9^	Pr, 2	Szabó et al. (2016)
	4.20±0.07 × 10^8^	LFP, 7	Lei et al. (2019)
Penicillin G, 2.9	3.30±0.30 × 10^8^	LFP, 7	Lei et al. (2019)
Penicillin V, 2.7	3.36±0.41 × 10^8^	LFP, 7	Lei et al. (2019)
7-Aminocephalosporanic acid, 2.6	2.29±0.11 × 10^8^	LFP, 7	Lei et al. (2019)
Cefotaxime, 2.7	4.91±0.16 × 10^8^	LFP, 7	Lei et al. (2019)
Cephalexin, 2.6, 6.4	5.06±0.29 × 10^8^	LFP, 7	Lei et al. (2019)
Cefaclor, 1.6	3.68±0.09 × 10^8^	LFP, 7	Lei et al. (2019)
*Benzenesulfonates and trimethoprim*			
Benzenesulfonic acid, 2.55	<10^5^	Pr, 7	Hasegawa and Neta (1978)
*p*-Cumenesulfonate, 1.34	9.4 × 10^9^	Pr, 6.5	Osiewala et al. (2013)
*p*-Styrene sulfonate	3.1 × 10^8^	Pr, 1	Bhardwaj et al. (2001)
Sulfacetamide, 5.4	8.0 × 10^8^	Pr, 2.0	Sabharwal et al. (1994)
Sulfanilamide, 2.3, 10.2	4.32±0.12 × 10^8^	LFP, 7	Lei et al. (2019)
Sulfadimethoxine, 2.1, 6.1	4.46±0.50 × 10^8^	LFP, 7	Lei et al. (2019)
Sulfadiazine, 2.1, 6.9	4.27±0.37 × 10^8^	LFP, 7	Lei et al. (2019)
Sulfamethazine 2.1, 7.5,	4.85±0.28 × 10^8^	LFP, 7	Lei et al. (2019)
Sulfamethoxazole, 1.6, 5.7	4.72±0.39 × 10^8^	LFP, 7	Lei et al. (2019)
Sulfathiazole, 2, 7.2	5.08±0.36 × 10^8^	LFP, 7	Lei et al. (2019)
Sulfapyridine, 2.2, 8.5	3.82±0.68 × 10^6^	Comp., 5	Liu et al. (2020)
1-Naphthylamine-4-sulphonic acid	7 × 10^7^	Pr, 1	Dwibedy et al. (2001)
Trimethoprim, 3.2, 7.1	2.4±0.3 × 10^9^	LFP, 5.5–6	Jasper et al. (2016)
	1.88±0.02 × 10^9^	LFP, 7	Lei et al. (2019)
*Fluoroquinolones*			
Ciprofloxacin, 6.1, 8.7	<5 × 10^7^	LFP, 5.5–6	Jasper et al. (2016)
	2.19±0.08 × 10^8^	LFP, 7	Lei et al. (2019)
Enrofloxacin, 6.1, 7.7	3.27±0.15 × 10^8^	LFP, 7	Lei et al. (2019)
Flumequine, 6.3	9.4±0.7 × 10^7^	LFP, 7	Lei et al. (2019)
Ofloxacin, 6.2, 8.1	3.48±0.39 × 10^8^	LFP, 7	Lei et al. (2019)
*Tetracyclines and tylosin*			
Tetracycline, 3.32, 7.8, 9.6	1.18±0.08 × 10^9^	LFP, 7	Lei et al. (2019)
Oxytetracycline, 3.1, 7.4, 8.9	9.36±0.29 × 10^8^	LFP, 7	Lei et al. (2019)
Doxycycline, 3.0, 8.0, 9.2	1.14±0.07 × 10^9^	LFP, 7	Lei et al. (2019)
Chlorotetracycline, 3.3, 7.6, 9.6	8.50±0.49 × 10^8^	LFP, 7	Lei et al. (2019)
Tylosin, 7.73	4.6±0.3 × 10^7^	LFP, 7	Lei et al. (2019)

7-Aminocephalosporanic acid may be regarded as the core part of the cephalosporin β-lactam antibiotics (Lei et al. 2019). This model compound reacts with *k*_Cl2•-_ = 2.29±0.11 × 10^8^ M^-1^ s^-1^. The rate constants of the cephalosporin antibiotics in the table are just slightly higher than this value suggesting reaction predominantly with the 7-aminocephalosporanic acid part. All values measured for β-lactams are in the same order of magnitude as measured for dimethyl- and diethyl sulfide (3.0 × 10^8^ and 4.7 × 10^8^ M^-1^ s^-1^, respectively, Bonifacic and Asmus 1980).

### Benzenesulfonates and trimethoprim

In benzenesulfonates a –SO_2_R group with highly oxidized sulfur atom is attached to a benzene ring. These molecules have good water solubility. They are used for numerous purposes including solubilisation or serve as starting molecules for synthesis of many drugs. Benzene sulfonic acid (Scheme 11S) is the basic molecule of the so-called sulfa drugs, sulfacetamide is the simplest antibiotic of the group, *p*-cumenesulfonate may serve as a model for sulfonate type surfactants, while *p*-styrene sulfonate is used to produce ionic polymers. Benzenesulfonic acid practically does not react with Cl_2_^•-^ due to the presence of the strong electron withdrawing sulfonate group (Hasegawa and Neta 1978). Osiewala et al. (2013) published an unexpectedly high value for the molecule which has a cumene group in *para* position (*p*-cumenesulfonate, 9.4 × 10^9^ M^-1^ s^-1^). Cl_2_^•-^ is suggested to react by electron transfer from aromatic ring and also by abstracting the tertiary H-atom of the cumene group. In the latter reaction benzyl type radical forms. The intermediate in reaction with a water molecule transforms to hydroxycyclohexadienyl radical.

Most of sulfa drugs have two p*K*_a_ values, the first acid-base dissociation occurs at pH 2–3 at the NH_2_ group attached to the benzene ring. The second one is at the –SO_2_–NH–R unit (–NH-  -N^-^- + H^+^) (Babic et al. 2007). In acidic solutions of sulfacetamide the transient formed in Cl_2_^•-^ reaction (and also in reactions of other one-electron oxidants) was inferred to be the radical cation, *k*_Cl2•-_ is 8.0 × 10^8^ M^-1^ s^-1^ (Sabharwal et al. 1994).

Lei et al. (2019) published *k*_Cl2•-_ ≈ 5 × 10^8^ M^-1^ s^-1^ for several sulfonamide antibiotics. At the pH of their measurements the molecules were in the neutral/anionic form. It is assumed that there is a common reaction center in these molecules. It should be noted that the rate constants of individual sulfonamides were very close to each-other also in CO_3_^•-^ reactions (~10^8^ M^-1^ s^-1^, Jasper and Sedlak 2013; Wols et al. 2014; Zhang et al. 2016). It is surprising that Liu et al. (2020) published lower value for the Cl_2_^•-^ + sulfapyridine reaction, 3.82±0.68 × 10^6^ M^-1^ s^-1^, as measured for other sulfonamide antibiotics.

Trimethoprim as antibiotic often used in combination with sulfa drugs, mainly with sulfamethoxazole. Jasper et al. (2016) and Lei et al. (2019) published similar high rate constant values with an average of 2.1 × 10^9^ M^-1^ s^-1^. In reaction with SO_4_^•-^ a rate constant of 7.7 × 10^9^ M^-1^ s^-1^ was measured while the reaction with CO_3_^•-^ proceeded with rate constant of 3.4 × 10^7^ M^-1^ s^-1^ (Zhang et al. 2015).

### Fluoroquinolones

Fluoroquinolones (Scheme 12S), ciprofloxacin, enrofloxaxin, flumequine and ofloxacin are used to treat both human and veterinary diseases caused by both Gram positive and Gram negative bacteria. These molecules contain a central fluoroquinolone unit and, in ciprorofloxacin, enrofloxaxin and ofloxacin a piperazine ring is attached to the benzene ring. Flumenique has three condensed rings. Ciprofloxacin, enrofloxacin, and ofloxacin at pH 7 are zwitterions with positive charge on the piperazine ring and negative charge on the carboxyl group. In ciprofloxacin the carboxyl group deprotonates with p*K*_a_ 6.1 and the deprotonation of the secondary nitrogen atom occurs with p*K*_a_ 8.7 (Jiang et al. 2016). In the experiments of Lei et al. (2019) ciprofloxacin was a zwitterion, while in the experiments of Jasper et al. (2016) it was a cation. The *k*_Cl2•-_ values were found to be highly different, 2.19±0.08 × 10^8^ and <5 × 10^7^ M^-1^ s^-1^, respectively. In the reactions of enrofloxacin and ofloxacin Lei et al. (2019) measured similar *k*_Cl2•-_ values as in ciprofloxacin reaction. The value of Lei et al. (2019) determined for flumequine is smaller, 9.4±0.7 × 10^7^ M^-1^ s^-1^, than that determined for the other three fluoroquilonones. Flumequine does not have piperazine ring. Jasper et al. (2016) in reaction of the strong one-electron oxidant SO_4_^•-^ suggested radical attack on this ring.

### Tetracycline antibiotics and tylosin

The four fused ring tetracycline antibiotics (Scheme 13S) exhibit wide range of activity against Gram positive and Gram negative bacteria and several classes of parasites. At pH 7 they are neutral molecules or zwitterions (Babic et al. 2007). Their *k*_Cl2•-_ values fall in narrow range with an average of 1.03±0.16 × 10^9^ M^-1^ s^-1^ (Lei et al. 2019). The macrolide antibiotic tylosin inhibits bacteria by binding to the 50S ribosome and inhibiting protein synthesis. The acivity spectrum is limited primarily to Gram positive aerobic bacteria. Tylosin reacts with *k*_Cl2•-_ of 4.6±0.3 × 10^7^ M^-1^ s^-1^ (Lei et al. 2019).

## Molecules with nitrogen atom(s) in the ring

3-Hydroxy pyridine at pH 2 is protonated, and as such has low reactivity toward Cl_2_^•-^ (<1.6 × 10^6^ M^-1^ s^-1^, Table 6, Scheme 14S). At pH 6.8, it is neutral (or a zwitterion with negative charge on oxygen (O^-^) and positive charge on nitrogen (NH^+^)). In the reaction, the semioxidized form is produced with *k*_Cl2•-_ = 1.44 × 10^8^ M^-1^ s^-1^ (Naik and Moorthy 1991b). 2-Hydroxy pyridine undergoes tautomerism to give 2-pyridone (a carbonyl compound). Pyridones are still aromatics as the lone pair of electrons on nitrogen can be delocalized into the ring. The *k*_Cl2•-_ of 2-hydroxy pyridine, 1.31 × 10^8^ M^-1^ s^-1^, is close to the value measured for 3-hydroxy pyridine (Naik and Moorthy 1991a). The rate constants of Cl_2_^•-^ reactions with 2- and 4-mercaptopyridines are in the 10^9^ M^-1^ s^-1^ range (Naik and Kishore 2002; Kishore et al. 2002).

**Table 6:** Molecules with nitrogen atom(s) in the ring

Compound, p*K*_a_	*k*_Cl2•-_, M^-1^ s^-1^	Method, pH	Literature
2-Hydroxy pyridine, −1.0, 9.97	1.31 × 10^8^	Pr, 2.5	Naik and Moorthy (1991a)
3-Hydroxy pyridine, 4.86, 8.72	1.44 × 10^8^	Pr, 6.8	Naik and Moorthy (1991b)
	<1.6 × 10^6^	Pr, 2	
2-Mercaptopyridine, −1.7, 9.97	3.0 × 10^9^	Pr, 1	Naik and Kishore et al. (2002)
4-Mercaptopyridine, 1.43	1 × 10^9^	Pr, 0	Kishore et al. (2002)
Imidazole, 7.1, 12.7	1.82±0.17 × 10^8^	LFP, 7	Lei et al. (2019)
Dimetridazole, 2.8	8.4±0.5 × 10^7^	LFP, 7	Lei et al. (2019)
Metronidazole, 2.6	1.24±0.08 × 10^8^	LFP, 7	Lei et al. (2019)
Ornidazole, 2.7	9.3±0.5 × 10^7^	LFP, 7	Lei et al. (2019)
Ronidazole, 1.3	1.55±0.08 × 10^8^	LFP, 7	Lei et al. (2019)
Pyrimidine	<10^6^	LFP, 7	Lei et al. (2019)
2,4-Dimethoxypyrimidine	3.0±0.2 × 10^7^	LFP, 7	Lei et al. (2019)
Purine, 2.4, 8.9	5.2±0.8 × 10^8^	FP, 7	Takahashi et al. (1985)
Xanthine, 7.7	1.85±0.11 × 10^8^	LFP, 7	Lei et al. (2019)
Caffeine, 10.4	9.28±0.52 × 10^8^	LFP, 7	Lei et al. (2019)
Theophylline, 8.8	8.78±0.34 × 10^8^	LFP, 7	Lei et al. (2019)
Adenine, 4.1, 9.8	<5 × 10^6^	Pr, 2.7	Ward and Kuo (1968)
Guanine, 3.3, 9.2	8.1 × 10^7^	Pr, 2.3	Ward and Kuo (1968)
Carbamazepine, 13.9	<5 × 10^7^	LFP, 5.5–6	Jasper et al. (2016)
	4.3±0.3 × 10^7^	LFP, 7	Lei et al. (2019)
Primidone, 12.3	1.58±0.02 × 10^8^	LFP, 7	Lei et al. (2019)
2-Thiouracil	3.2 × 10^9^	Pr, 1	Prasanthkumar et al. (2012a)
4-Thiouracil	2.7 × 10^9^	Pr, 1	Prasanthkumar et al. (2012b)

Imidazole, a diazole type aromatic heterocycle, shows high reactivity in one-electron oxidation: the rate constants with SO_4_^•-^ and Cl_2_^•-^ are 5.3 × 10^9^ M^-1^ s^-1^ and 1.82±0.17 × 10^8^ M^-1^ s^-1^, respectively (Steenken 1989; Lei et al. 2019). The nitroimidazole type of antibiotics, dimetridazole, metridazole, ornidazole and ronidazole, have central nitroimidazole units, and substituents are added to one of the N- and C-atoms. They react with average *k*_Cl2•-_ of 1.14±0.32 × 10^8^ M^-1^ s^-1^ (Lei et al. 2019). This value is between the rate constants determined in CO_3_^•-^ and SO_4_^•-^ reactions: 3.34±0.47 × 10^7^ and 2.72±0.41 × 10^9^ M^-1^ s^-1^, respectively (Wojnárovits and Takács 2019; Wojnárovits et al. 2020). The trend of the average values reflects the trend of reduction potentials of the oxidants. The similar values within the group suggest that these radicals attack the same parts of the molecules, most probably the imidazole ring. The *k*_Cl2•-_ values measured for imidazole and the nitroimidazoles practically coincide.

In reaction with one-electron oxidants, among them also with Cl_2_^•-^, pyrimidine shows low reactivity (Steenken 1989; Lei et al. 2019). The reactivity is increasing when two electron donating methoxy groups are attached to the ring in 2,4-dimethoxypyrimidine. The basic structure of the bicyclic compounds in Table 7 is purine. Purine is a heterocyclic aromatic compound with a pyrimidine ring fused to an imidazole ring. Adenine and guanine nucleobases react with Cl_2_^•-^ with low rate constants of <5 × 10^6^ and 8.1 × 10^7^ M^-1^ s^-1^, respectively (Ward and Kuo 1968): the pyrimidine ring deactivates the imidazole ring against radical attack. In xanthine, caffeine, and theophylline (stimulants in coffee, tea, and cola) the pyrimidine ring has no aromatic character, the *k*_Cl2•-_ values are in the 10^8^ M^-1^ s^-1^ range (Lei et al. 2019).

**Table 7:** Miscellaneous compounds

Compound, p*K*_a_	*k*_Cl2•-_, M^-1^ s^-1^	Method, pH	Literature
Tetramethylammonium cation	<10^3^	Pr, 1	Bobrowski (1980)
Tetraethylammonium cation	6 × 10^3^	Pr, 1	Bobrowski (1980)
Tetrapropylammonium cation	8 × 10^4^	Pr, 1	Bobrowski (1980)
Tetrabutylammonium cation	3 × 10^4^	Pr, 1	Bobrowski (1980)
CTACl	1.2 × 10^7^	Pr, 2	Patterson et al. (1972)
Vinylbenzyltrimethyl-ammonium chloride	2.3 × 10^8^	Pr, 1.2	Kumar et al. (2003)
NaLS	3.9 × 10^6^	Pr, 2	Patterson et al. (1972)
Igepal CO-730	2.1 × 10^8^	Pr, 2	Patterson et al. (1972)
Clenbuterol, 9.7	5.54±0.14 × 10^8^	LFP, 7	Lei et al. (2019)
Mabuterol, 13.4	3.48±0.27 × 10^8^	LFP, 7	Lei et al. (2019)
Salbutamol, 10.1	3.02±0.20 × 10^8^	LFP, 7	Lei et al. (2019)
Terbutaline, 8.8, 10.1	1.20±0.07 × 10^9^	LFP, 7	Lei et al. (2019)
Iopromide, 10.6	2.05±0.10 × 10^9^	LFP, 7	Lei et al. (2019)
BiNPO_4_H, 6.5	3.2±1.0 × 10^7^	Pr, 2.7	Shoute (1997)
Metformin, 12.4	2.1±0.1 × 10^7^	LFP, 7	Lei et al. (2019)
Sucralose, 12.5	<1 × 10^6^	LFP, 7	Lei et al. (2019)
Hyaluronan	6.9 × 10^6^	Pr, 1	Al-Assaf et al. (2006)
Ascorbic acid, 4.2 11.6	6.8 × 10^8^	Pr, 2	Schöneshöfer (1972)
	6.0 × 10^8^	Pr, 2	Redpath and Willson (1973)
2,3-Dihydroxy-2-propenal	1 × 10^9^		Horii et al. (1985)
Microcystin-LR	5.58±0.42 × 10^7^	LFP, 3.9	Zhang et al. (2019)

Carbamazepine is used in cases of neuropathic disorders. Jasper et al. (2016) suggested a *k*_Cl2•-_ of <5 × 10^7^ M^-1^ s^-1^, while the value of Lei et al. (2019) is 4.3±0.3 × 10^7^ M^-1^ s^-1^. In SO_4_^•-^ reaction a rate constant of 1.92 × 10^9^ M^-1^ s^-1^ was determined (Matta et al. 2011), whereas in CO_3_^•-^ reaction 3.3±1 × 10^6^ M^-1^ s^-1^ (average of 4 values) was found (Wojnárovits et al. 2020). The *k*_Cl2•-_ of pirimidone (epilepsy compound) was determined to be 1.58±0.02 × 10^8^ M^-1^ s^-1^.

In 2-thiouracil and 4-thiouracil the S-atom essentially determines their reactivity with Cl_2_^•-^: the rate constants are high 3.2 × 10^9^ and 2.7 × 10^9^ M^-1^ s^-1^, respectively, and three-electron bonded dimer radical cation intermediates were observed as it is typical for sulfur compounds (Prasanthkumar et al. 2012a, b).

## Miscellaneous compounds

Tetramethyl-, tetraethyl-, tetrapropyl, and tetrabutylammonium ions have low reactivity with Cl_2_^•-^ (Scheme 15S, Bobrowski 1980). The reactivity is increasing with the increasing number of C-H bonds in the molecule. The values (10^3^-10^4^ M^-1^ s^-1^) are in good agreement with those of the other aliphatic compounds which undergo H-abstraction. Cetyltrimethylammonium chloride (CTACl) is used as a cationic surfactant (Table 7). The molecule has 14 –CH_2_- units, therefore, many possibilities for H-abstraction. The rate constant, 1.2 × 10^7^ M^-1^ s^-1^, is much higher than that of the previously mentioned trialkylammonium ions (Patterson et al. 1972). In vinyltrimethylammonium chloride, the reaction takes place on the styrene part of the molecule forming radical cation (2.3 × 10^8^ M^-1^ s^-1^, Kumar et al. 2003).

Table 7 lists also the rate constant of the cationic surfactant sodium dodecyl sulfate (NaLS) and the neutral nonylphenol ethoxylate (Igepal CO-730) surfactant (3.9 × 10^6^ and 2.1 × 10^8^ M^-1^ s^-1^, respectively, Patterson et al. 1972). The *k*_Cl2•-_ for the latter is much higher due to the presence of the –O-CH- moiety, the rate constant is similar to that of anisole, 1.62±0.09 × 10^8^ M^-1^ s^-1^ (Lei et al. 2019). When the concentrations of surfactants were above critical micelle concentrations (CMC) the rate constants decreased considerably due to aggregation.

Clenbuterol, mabuterol, salbutamol, and terbutaline are used by patients with breathing disorders. These compounds have the same –CH(OH)-CH_2_-NH-C(CH_3_)_3_ side on a benzene ring. Similarly, to the previously discussed blood pressure regulators, they also have a secondary amine in the chain. In clenbuterol and mabuterol the ring is deactivated by chlorine or trifluoromethyl groups. In salbutamol and terbutaline activating –OH or –CH_2_-OH groups are attached to the rings. Despite the activating or inactivating groups the *k*_Cl2•-_ values are close to each-other, they are in the 3 × 10^8^–1.2 × 10^9^ M^-1^ s^-1^ range (Lei et al. 2019). Cl_2_^•-^ probably attacks the amine containing side-chain, which is common in all of them. The *k*_Cl2•-_ values are one order of magnitude higher as measured for CO_3_^•-^, and smaller by one order of magnitude as determined for SO_4_^•-^ reactions (Wojnárovits and Takács 2019; Wojnárovits et al. 2020)

Due to the three heavy iodine atoms, iopromide is used as a contrast material in the medical practice; *k*_Cl2•-_ is high, 2.05±0.10 × 10^9^ M^-1^ s^-1^ (Lei et al. 2019). Cl_2_^•-^ reacts with R)-(−)-1,1‘-binaphthyl-2,2‘-diyl hydrogen phosphate (BiNPO_4_H_)_, used as model compound of soluble aromatic molecules, in electron transfer oxidation (Shoute 1997). Metformin is used for the treatment of type 2 diabetes. This simple molecule reacts with *k*_Cl2•-_ = 2.1±0.1 × 10^7^ M^-1^ s^-1^ (Lei et al. 2019). Sucralose, a widely used artificial sweetener, in reaction with Cl_2_^•-^ has low reactivity, *k*_Cl2•-_ < 1 × 10^6^ M^-1^ s^-1^ (Lei et al. 2019). The radical is expected to react with sucralose in H-abstraction, such reactions are generally slow as the example of aliphatic alcohols shows. Small rate constant was measured also in SO_4_^•-^ reaction (Wojnárovits and Takács 2019). The value published for the hyaluronan, nonsulfated glycosaminoglycan type biopolymer, was also small, *k*_Cl2•-_ = 6.9 × 10^6^ M^-1^ s^-1^ Al-Assaf et al. (2006). Cl_2_^•-^ is assumed to attack at the N-acetyl function initiating chain type depolymerisation.

The reaction of ascorbic acid with Cl_2_^•-^ (and other one-electron oxidants) at pH 2 gives ascorbic acid radical, *k*_Cl2•-_ = 6.4 × 10^8^ M^-1^ s^-1^ (average, Schöneshöfer 1972; Redpath and Willson 1973). Microcystin-LR, a cyclic heptapeptide containing seven amino acids, is a strong cyanotoxin. The rate constant measured for its reaction with Cl_2_^•-^ is unexpectedly small for such a large molecule: 5.58±0.42 × 10^7^ M^-1^ s^-1^ (Zhang et al. 2019).

### Dye cations

The dye cations listed in Table 8 (Scheme 16S), with the exception of the four textile dyes, are principally used for biological purposes in staining processes or as redox indicators (e.g., in titration). In one-electron oxidation an electron is removed from the aromatic system and the formed conjugated semioxidized forms have intense light absorption in the visible range with molar absorption coefficient going up to several times 10^4^ M^-1^ cm^-1^.

**Table 8:** Dye cations

Compound, p*K*_a_	*k*_Cl2•-_, M^-1^ s^-1^	Method, pH	Literature
Thionine, 0.3, 11	3.3 × 10^9^	Pr, 1.7	Kishore et al. (1987)
Methylene blue, 3.1	8±0.5 × 10^9^	Pr, 1.7	Kishore et al. (1989)
Toluidine blue, 2.4, 11.6	3.4 × 10^9^	Pr, 1.8	Mahadevan et al. (1990)
Promethazine, 9.6	5 × 10^9^	Pr, 1–2	Willson (1973)
Chlorpromazine, 9.6	5 × 10^9^	Pr, 1–2	Willson (1973)
Acriflavine cation	~4 × 10^9^	Pr	Prütz and Land (1970)
Acridine-1,8-dione	4.0 × 10^9^	Pr, 1	Mohan et al. (1996)
Riboflavine, 6.97	2.1 × 10^10^	Pr, 1.7	Kishore et al. (1991)
Neutral red, 6.7	7.5 × 10^9^	Pr, 1.8	Guha et al. (1993)
Safranine T, 5.8	5.5 × 10^9^	Pr, 2	Guha et al. (1992)
Reactive Blue 81	8.98 × 10^8^	Pr, 3	Ledakowicz et al. (2012)
Acid Red 27	6.51 × 10^8^	Pr, 3	Ledakowicz et al. (2012)
Reactive Red-120	1 × 10^8^	Pr,	Paul et al. (2010)
Orange II	3.2±1.0 × 10^7^	LFP, 2.8	Kiwi et al. (2000)

Thionine, methylene blue, and toluidine blue have the same phenothiazine core. In thionine in 2,8-positions there are NH_2_ groups, in methylene blue N(CH_3_)_2_ groups are in these positions, in toluidine NH_2_, N(CH_3_)_2_ and CH_3_ groups are added to the core. At the pH of measurements (~1.7) these dyes are in single or double ionized forms. Cl_2_^•-^ reacts with these cations with *k*_Cl2•-_ values of 3.3 × 10^9^, 3.4 × 10^9^, and 8±0.5 × 10^9^ M^-1^ s^-1^, respectively (Kishore et al. 1987, 1989; Mahadevan et al. 1990). The reduction potential of thionine is likely to be close to but more positive than that of the N_3_^•^/N_3_^-^ couple, i.e., 1.33 V *vs*. NHE (Kishore et al. 1987). Those of methylene blue and toluidine blue were calculated as 1.25 and 1.16 V, respectively, *vs*. NHE (Kishore et al. 1989; Mahadevan et al. 1990). The higher reduction potential of the Cl_2_^•-^/2Cl^-^ couple than the couples of the semioxidized/nonoxidized forms of dyes gives explanation for the high values. Promethazine and chlorpromazine are used for medical purposes as antipsychotic medications. They react with *k*_Cl2•-_ of 5 × 10^9^ M^-1^ s^-1^ (Willson 1973).

Acriflavine (ACR) is an acridine type dye which exhibits chemiluminescence after reaction with free radicals. Prütz and Land (1970) estimated a *k*_Cl2•-_ of ~4 × 10^9^ M^-1^ s^-1^. ACR reacted also with (SCN)_2_^•-^ and Br_2_^•-^, but the reaction with I_2_^•-^ is very slow. The reduction potential of the semioxidized ACR^•^/ACR couple is expected to be around 1 eV *vs*. NHE. One-electron oxidants readily oxidize acridine-1,8-dione which has gained importance as a laser dye. They remove the electron from the N-atom (*k*_Cl2•-_ = 4 × 10^9^ M^-1^ s^-1^, Mohan et al. 1996).

The Cl_2_^•-^ reaction with riboflavine (Rf) by removing an electron from the extended conjugated system is an equilibrium process. Using the equilibrium constant the reduction potential of Rf^+^/Rf couple has been evaluated to be 2.28 V *vs*. NHE (Kishore et al. 1991). *k*_Cl2•-_ was reported to be 2.1 × 10^10^ M^-1^ s^-1^. This value is unrealistically high, higher than the diffusion controlled limit (c.a. 7.0 × 10^9^ M^-1^ s^-1^, Wojnárovits and Takács 2016). Neutral red, a phenazine type dye, is used for staining in histology (staining lysosomes red) and as a pH indicator. Guha et al. (1993) published high rate constants for its reaction with Cl_2_^•-^: 7.5 × 10^9^ M^-1^ s^-1^.

High value (5.5 × 10^9^ M^-1^ s^-1^) was published also for the reaction of safranine T cation (Guha et al. 1992). Safranines are azonium compounds of the symmetrical 2,8-dimethyl-3,7-diaminophenazine. The reduction potential in the one-electron oxidation is 1.15 V *vs*. NHE. Safranine T is as a biological stain used in histology and cytology.

Dying solutions usually contain high Cl^-^concentrations. Cl^-^ (depending on pH) removes a large part of ^•^OH during advanced oxidation processes transforming them to Cl_2_^•-^. Ledakowicz et al. (2012) measured the rate constants of Cl_2_^•-^ reactions with Reactive Blue 81 and Acid Red 27 azo dyes as 8.98 × 10^8^ and 6.51 × 10^8^ M^-1^ s^-1^, respectively. These values are much smaller than those found for ^•^OH reactions (1.98 × 10^9^ and 9.17 × 10^9^ M^-1^ s^-1^, respectively). *k*_Cl2•-_ = 1 × 10^8^ and 3.2±1.0 × 10^7^ M^-1^ s^-1^, respectively, were published for the reactions of Reactive Red-120 and Orange II (Kiwi et al. 2000; Paul et al. 2010).

## Mechanism of electron transfer

It is apparent from the discussion of Cl_2_^•-^ reactions with individual compounds that the authors mostly suggested electron transfer as a probable mechanism. Previously in connection with the possibility of reaction we often referred to the differences between the reduction potentials of the Cl_2_^•-^/2Cl^-^ couple and those of the one-electron oxidized/nonoxidized organic molecule couples. The higher, or at least similar reduction potential value of the former couple to that of the latter one gives only a possibility for the transfer. In the Supplementary Material, using the Marcus theory, we discuss the relation between rate constant values and the reduction potential differences for the reactions of phenols and anilines with Cl_2_^•-^, CO_3_^•-^, and SO_4_^•-^ one-electron oxidants. The analysis shows that there is a correlation between the two quantities. However, other effects, e.g., solvent reorganization around the charged species and steric arrangement of the reactive parts of molecules, also strongly influence the rate constant values.

## Concluding remarks

1. About 400 rate constants for ~300 compounds were collected from the literature, for most compounds only one value was published. Therefore, we had little possibilities to compare the values measured for the same compound, eventually by different techniques. When there were possibilities for comparison the *k*_Cl2•-_ values often differed considerably. This may be due to the rather complex reaction systems; it is difficult to find optimal conditions to measure *k*_Cl2•-_. The large differences, in some cases are due to improper handling the transient data.
2. Practically all rate constants were measured by transient techniques, by (laser) flash photolysis or pulse radiolysis following the decay of Cl_2_^•-^. Rate constant determination by competitive technique was reported in few publications.
3. The measured *k*_Cl2•-_ values span over at least 6 orders of magnitude. The highest values are around the diffusion controlled limit (~7.0 × 10^9^ M^-1^ s^-1^).
4. Most of the information about the reaction mechanism is coming from indirect sources. Actually, the authors rarely analysed the absorption spectra in transient measurements.
5. Cl_2_^•-^ can abstract an H-atom from aliphatic compounds (e.g., methanol, ethanol, 2-propanol) with rate constants between 10^3^ and 10^5^ M^-1^ s^-1^. The *k*_Cl2•-_ values were shown to relate to the energy of the bond broken during H-abstraction.
6. Olefin compounds react with Cl_2_^•-^ by three orders of magnitude more rapidly (10^5^-10^8^ M^-1^ s^-1^) than the saturated analogs: the reactions with olefins occur with Cl-addition. Addition to the aromatic ring may also take place with *k*_Cl2•-_ of <10^7^ M^-1^ s^-1^, but direct oxidation of the ring by electron transfer seems to be the predominant pathway.
7. The reactions with phenols, anilines, sulfur compounds (with sulfur atom in lower oxidation stste), molecules with conjugated electron systems are suggested to take place with electron transfer (10^7^–10^9^ M^-1^ s^-1^). However, it is noted in some works that electron transfer and Cl_2_^•-^ addition to a double with eliminations of two Cl^-^ may give the same result. The rate constant is high when the reduction potential of the one-electron oxidized species/molecule couple is well below that of the Cl_2_^•-^/2Cl^-^ couple. However, other effects, e.g., solvent reorganization around the charged species and steric arrangement of the reactive parts of molecules, also strongly influence the values.
8. The rate constant values measured for CO_3_^•-^, Cl_2_^•-^, and SO_4_^•-^ one-electron oxidants increase in this order; it is the order of increasing one-electron reduction potentials.

## Supplementary Information

ESM 1 (PDF 1.46 mb)
