# Supplementary material for: Rate constants of dichloride radical anion reactions with molecules of environmental interest in aqueous solution: a review
Source: Environ Sci Pollut Res Int. 2021 Jun 4;28(31):41552–75. doi: 10.1007/s11356-021-14453-w (PMC8354983; doi:10.1007/s11356-021-14453-w)
Supplement: Supplementary file 1 — (PDF 1.46 mb) [file 11356_2021_14453_MOESM1_ESM.pdf]

Supplementary Material

Rate constants of dichloride radical anion reactions with molecules of environmental interest in aqueous solution, a review

László Wojnárovits, *Erzsébet Takács
Radiation Chemistry Department, Institute for Energy Security and Environmental Safety, Centre for Energy Research, ELKH, H-1121 Budapest, Konkoly-Thege Miklós út 29-33, Hungary

This Supplementary Material contains: 1. reduction potentials of several inorganic radicals (Table 1S), 2. reduction potentials of larger number of phenol and aniline compounds, the rate constants of their reactions with $Cl_2^{\bullet-}$, $CO_3^{\bullet-}$ and $SO_4^{\bullet-}$ radicals (Tables 2S-4S), and the relation between the two quantities using the Marcus plot (Figure 1S) together with evaluation of the correlation. It also contains: 3. the chemical structure of compounds whose rate constants are discussed in the manuscript (Schemes 1S-16S).

1. Redox properties of inorganic radicals

The standard reduction potential of the $Cl_2^{\bullet-}/2Cl^-$ couple has been reported to be between 2.1 and 2.3 V *vs*. NHE (Wardman, 1989), in the review the frequently referred value $E^o(Cl_2^{\bullet-}/2Cl^-)$ = 2.1 is used. Table 1 lists a number of one-electron oxidants with reduction potentials ranging from 2.43 V to 0.934 V.

Table 1S. Standard reduction potentials of inorganic one-electron oxidizing couples.

Couple	E^o, V	Reference
$SO_4^{\bullet-}/SO_4^{2-}$	2.43	Armstrong et al., 2015
$Tl^{\bullet 2+}/Tl^+,H^+$	2.22	Schwartz et al., 1974
$Cl_2^{\bullet-}/2Cl^-$	2.1	Wardman, 1989
$CO_3^{\bullet-}/CO_3^{2-}$	1.78 pH 7.0	Arnold, 2014
$Br_2^{\bullet-}/2Br^-$	1.63	Armstrong et al., 2015
$N_3^{\bullet}/N_3^-$	1.33	Armstrong et al., 2015
$(SCN)_2^{\bullet-}/2(SCN)^-$	1.30	Armstrong et al., 2015
$I_2^{\bullet-}/2I^-$	1.04	Armstrong et al., 2015
$ClO_2^{\bullet}/ClO_2^-$	0.934	Armstrong et al., 2015

Among other methods (e.g., electrochemical techniques) the reactions with one-electron oxidants are also frequently used to determine the reduction potentials of inorganic and organic molecules. Based on a correlation between the efficiency of oxidation of a compound and the reduction potential of the radical, the standard potential for the one-electron oxidation/reduction of the compound investigated can be inferred. The one-electron reduction potential of organic molecules can be precisely calculated under special conditions when the reaction is reversible and equilibrium (1S) is established within a few microseconds (Wardman, 1989; Armstrong et al., 2015). $Cl_2^{\bullet-}$ was also used for such purpose (Kishore et al., 1991):

$$Cl_2^{\bullet-} + S \underset{k_b}{\overset{k_f}{\rightleftharpoons}} 2Cl^- + S^+ \qquad (1S)$$

where k_f and k_b are the rate constants of the forward and backward reactions at the equilibrium.

2. *Mechanism of electron transfer, evaluation based on the Marcus theory*

In the manuscript we often referred to the differences between the reduction potentials of the $Cl_2^{\bullet -}/2Cl^-$ couple and those of the one-electron oxidized/non-oxidized organic molecule couples. In Figure 1S we show the logarithms of rate constant values for the reactions of phenols and anilines with $Cl_2^{\bullet -}$, $CO_3^{\bullet -}$ and $SO_4^{\bullet -}$ one-electron oxidants ($k_{Cl2\bullet -}$, $k_{CO3\bullet -}$ or $k_{SO4\bullet -}$) as a function of reduction potential differences (ΔE). The $k_{CO3\bullet -}$ and $k_{SO4\bullet -}$ values were taken from our previous review papers (Wojnárovits and Takács, 2019; Wojnárovits et al., 2020), in several cases the values are averages of several determinations. The reduction potentials of organic couples were collected from a large number of papers, e.g., Steenken and Neta (1982), Wardman (1989), Lind et al. (1990), Jonsson et al. (1994), Li and Hoffman (1999), Roder et al. (1999) and Arnold (2014).

Between 10^6 and 10^8 M^{-1} s^{-1} the k values increase with ΔE, then around 10^8-10^9 M^{-1} s^{-1} the k values show a tendency to level off. To a large extent this is due to the diffusion limitation ($k_{diff} \approx 7 \times 10^8$ M^{-1} s^{-1}) which suppresses the rate constant range. In the figure the lines were calculated using equ. (2S) developed for electron transfer reactions (Marcus and Sutin, 1985). The magnitude of the rate constant values can be evaluated based on the Marcus theory.

$$k_{act} = Z \exp - \frac{\lambda^0}{4RT}\left[1 + \frac{\Delta G^0}{\lambda^0}\right] \qquad (2S)$$

$$k_{act} = \frac{k_{diff}\, k_{act}}{k_{diff} + k_{act}} \qquad (3S)$$

Z was taken, as usual 1×10^{11} M^{-1} s^{-1} (Armstrong et al., 1996; Meisel, 1975; Jonsson et al., 1993). The free energy difference was calculated using ΔE (kJ mol^{-1}) = $\Delta G^0 = F\, \Delta E$ (V, F is the Faraday's constant). k_{diff} and k_{act} stand for the diffusion and activation controlled rate constant. For the λ^0 reorganization energy values between 90 and 230 kJ mol^{-1} were used.

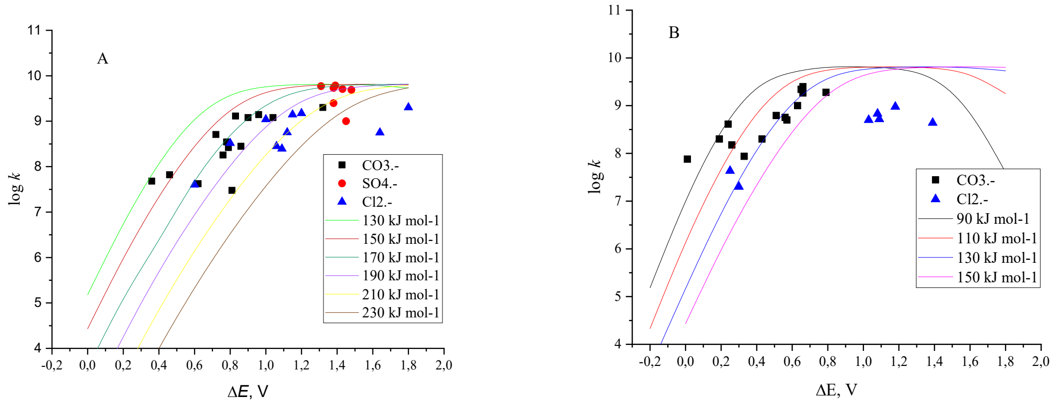

Figure 1S. Relation between the electron transfer rate constants and ΔE in cases of phenols (A) and anilines (B). The rate constant and the reduction potential values used in constructing the figures are collected in Tables 2S-4S.

In phenol reactions ($XPhO^{\bullet}/XPhO^{-}$ couple) the figure suggests λ^0 reorganization energy in the 150-190 kJ mol^{-1} range, for anilines ($PhNH_2^{\bullet +}/PhNH_2$) the suggested range is between 110-150 kJ mol^{-1}. In the reactions of phenoxyl radicals and phenolates generally smaller λ^0 values, 60-80 kJ mol^{-1} were found (Armstrong et al., 1996). In electron transfer between ($O_2/O_2^{\bullet -}$) and ($PhO^{\bullet}/PhO^{-}$) Jonsson et al. (1993) found λ^0 =155 kJ mol^{-1}.

According to the Marcus additivity relationship (Marcus, 1965) the reorganization energy is a composition of the self-exchange reactions of the reactants (λ_{se}), here we write the equation for $Cl_2^{\bullet -}$ reaction with phenolates:

$$\lambda^0 = ½ (\lambda_{se}) (Cl_2^{\bullet -}/2Cl^{-}) + ½ (\lambda_{se}) (XPhO^{\bullet}/XPhO^{-}) \quad (4S)$$

For organic couples Johnson et al. (1993) suggested a λ_{se} value of 62 kJ mol^{-1}. The λ_{se} value of the inorganic couples should be at least two times higher. This is probably due to the strong orientation of water molecules around the negatively charged inorganic ions. In $Cl_2^{\bullet -}$ reactions two Cl^{-} ions form requiring higher reorganization of the solvent molecules. This high reorganization energy also limits the rate constants. We note that the points belonging to $Cl_2^{\bullet -}$ reactions in Fig. 1S are shifted to higher ΔE values as compared to those of $CO_3^{\bullet -}$ reactions.

Table 2S. Reactions $Cl_2^{\bullet-}$. (In brackets the pH value is given)

Compound	$k_{Cl2\bullet-}$, M^{-1} s^{-1} (pH)	Reduction pot., V (pH)
Catechol	5.66×10^8 (7)[a)]	0.46 (7)[e)]
Dopamine	2×10^9(1.5)[b)]	0.70 (12)[f)]
Bisphenol A	5.82×10^8 (7)[a)]	0.77 (7)[f)]
Phenol	3.8×10^8 (1)[c)]	1.30 (1)[g)]
Hydroquinone	1.4×10^9 (1)[d)]	0.95 (1)[g)]
Triclosan	2.48×10^8 (7)[a)]	1.01 (12)[f)]
4-Hydroxybenzoic acid	1.5×10^9 (9.5)[d)]	0.90 (12)[e)]
4-Hydroxybenzoic acid	2.8×10^9 (7)[d)]	1.04 (7)[g)]
4-Methoxyphenol	1.1×10^9 (1)[d)]	1.1 (1)[g)]
4-Cyanophenol	4×10^7 (1)[d)]	1.50 (1)[g)]
Acetaminophen	4.4×10^8(7)[a)]	0.707 (7)[h)]
4-Toluidine	9.5×10^8 (7)[a)]	0.92 (12)[f)]
4-Chloroaniline	5.23×10^8 (7)[a)]	1.01 (12)[i)]
Aniline	6.79×10^8 (7)[a)]	1.02 (12)[f)]
Indomethacin	5.0×10^8 (7)[a)]	1.07 (12)[j)]
Carbamazepine	4.3×10^7 (7)[a)]	1.95 (12)[k)]
Acetanilide	2×10^7 (7)[d]	1.90 (12)[k)]

[a)]Lei et al, 2019, [b)]Dhiman and Naik, 2010, [c)] Average, this paper [d)] Hasegawa and Neta, 1978, [e)] Lind et al., 1990, [f)] Pavitt et al., 2017. [g)]Jovanovic et al., 1991, [h)] Thu et al., 2018, [i)] Jonsson et al., 1994, [j)] Sataraddi et al., 2014, [k)] Arnold, 2014

Table 3S. Reactions of $CO_3^{\bullet-}$. (In brackets the pH value is given)

Compound	$k_{CO3\bullet-}$, M^{-1} s^{-1} (pH)	Reduction pot., V (pH)
Hydroquinone	2×10^9 (7)[a)]	0.46 (7)[i)]
4-Methoxyphenol	1.2×10^9 (11)[b)]	0.54(12)[i)]
2,6-Dimethoxyphenol	1.4×10^9 (12)[b)]	0.62 (12)[j]
4-Methylphenol	1.2×10^9 (12)[b)]	0.68 (12)[i)]
2,4,6-Trimethylphenol	1.3×10^9 (12)[b)]	0.75 (12)[j)]

Phenol	2.6×10^8 (12)[c)]	0.79 (12)[i)]
4-Chlorophenol	3.5×10^8 (11)[e)]	0.80 (12)[i)]
4-Bromophenol	1.8×10^8 (12)[f)]	0.82 (12)[i)]
Bisphenol A	2.8×10^8 (8.8)[d)]	0.84 (8.8)[j)]
3-Methoxyphenol	5.1×10^8 (12)[b)]	0.86 (12)[j)]
Phenol	3×10^7 (7)[c)]	0.97 (7)[i)]
Triclosan	4.2×10^7 (8.8)[d)]	1.01 (12)[j)]
4-Cyanophenol	6.6×10^7 (11)[c)]	1.12 (12)[i)]
4-Nitrophenol	4.8×10^7 (12)[d)]	1.22 (12)[i)]
4-Methoxyaniline	1.9×10^9 (9)[g)]	0.79 (12)[k)]
N-Methylaniline	2.5×10^9 (11)[e)]	0.92 (12)[j)]
N,N-Dimethylaniline	1.85×10^9 (11)[e)]	0.92 (12)[j)]
N-Ethylaniline	2.2×10^9 (11)[e)]	0.93 (12)[j)]
4-Methylaniline	1.0×10^9 (11)[c)]	0.95 (12)[k)]
4-Chloroaniline	5×10^8 (9)[c)]	1.01 (12)[k)]
Aniline	5.62×10^8 (11)[c)]	1.02 (12)[k)]
4-Fluoraniline	6.2×10^8 (8.5)[h)]	1.07 (12)[j)]
4-Amiobenzoic acid	2×10^8 (9)[h)]	1.15 (12)[j)]
4-Aminobenzene sulfonate	8.7×10^7 (11)[e)]	1.25 (12)[j)]
4-Cyanoaniline	1.5×10^8 (11)[e)]	1.32 (12)[k)]
3,4-dichloroaniline	4.1×10^8 (11)[e)]	1.34 (12)[j)]
Ethyl-4-aminobenzoate	2×10^8 (8.5)[h)]	1.39 (12)[j)]
4-Nitroaniline	7.7×10^7 (11)[e)]	1.57 (12)[j)]

a) Huie et al., 1991, [b)] Stenman et al., 2003, [c)]Average, Wojnárovits et al., 2020, [d)] Huang et al., 2018, [e)] Canonica et al., 2005, [f)] Moore et al., 1977, [g)] Huang and Mabury, 2000; [h)] Elango et al., 1984, [i)] Lind et al., 1990, [j)] Pavitt et al., 2017, [k)]Jonsson et al., 1994

Table 4S. Reactions of $SO_4^{\bullet-}$. (In brackets the pH value is given)

Compound	$k_{SO4\bullet-}$, M^{-1} s^{-1} (pH)	Reduction pot., V (pH)
Bisphenol A	1.0×10^9 (7)[a]	0.77 (7)[e)]
p-Cresol	4.9×10^9 (5.8)[a)]	0.95 (5.8)[d)]
o-Cresol	5.1×10^9 (5.8)[a)]	1.00 (5.8)[d)]
m-Cresol	5.4×10^9(5.8)[a)]	1.05 (5.8)[d)]
4-Hydroxybenzoic acid	2.5×10^9 (7)[c)]	1.04 (12)[e)]
Phenol	6.1×10^9 (5.8)[a)]	1.04 (5.8)[d)]
4-Hydroxybenzaldehyde	5.9×10^9 (7)[b)]	1.12 (12)[e)]

a) Average, Wojnárovits and Takács, 2019, [b)] Geeta et al., 2001, [c)] Neta et al., 1977, [d)] Roder et al., 1999, [e)] Pavitt et al., 2017, [f)] Lind et al., 1990

3. *Chemical structures of compounds discussed*

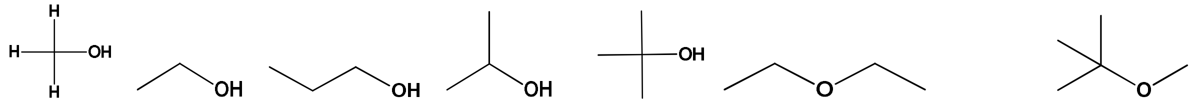

Methanol Ethanol 1-Propanol 2-Propanol *tert*-Butanol Diethylether Methyl-*tert*-butyl ether

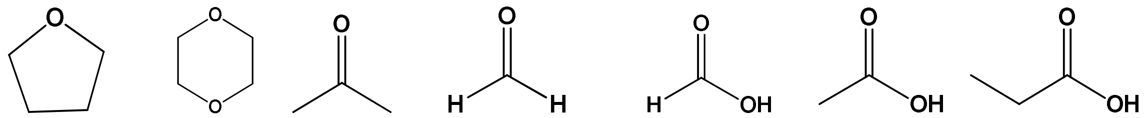

Tetrahydrofuran Dioxan Acetone Formaldahyde Formic acid Acetic acid Propionic acid

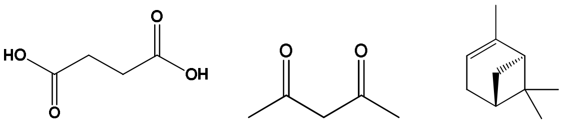

Succinic acid Acetylacetone α-Pinene

Scheme 1S. Small molecules of environmental interest

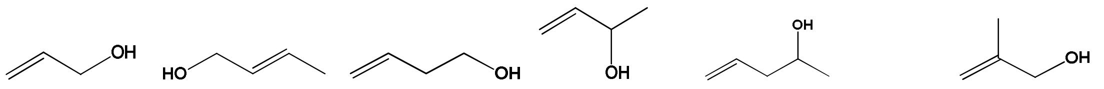

Allylalcohol 2-Buten-1-ol 3-Buten-1-ol 3-Buten-2-ol 4-Penten-2-ol 2-Methyl-2-propen-1-ol

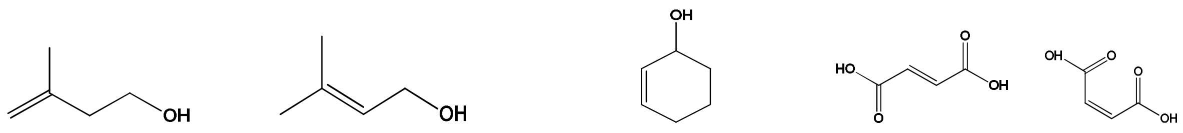

3-Methyl-3-buten-1-ol 3-Methyl-2-buten-1-ol 2-Cyclohexen-1-ol Fumaric acid Maleic acid

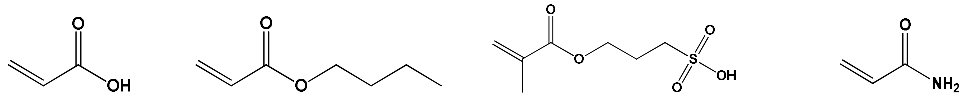

Acrylic acid n-Butyl acrylate 3-Sulfo propylmethacrylate Acrylamide

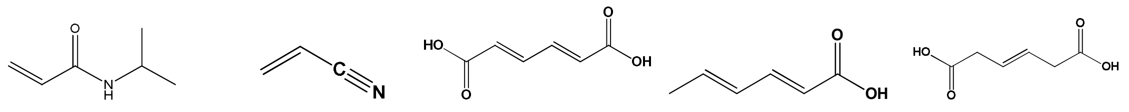

N-Isopropyl acrylamide Acrylonitrile Muconic acid Sorbic acid 3-Hexenedioic acid

Scheme 2S. Unsaturated alcohols and carboxylic acids

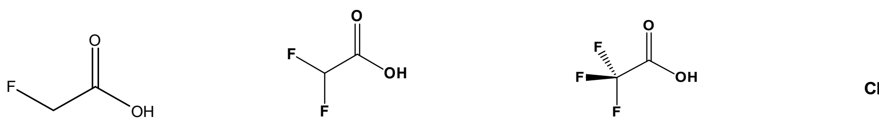

Monofluoroacetic acid Difluoroacetic acid Trifluoroacetic acid Monochloroacetic acid

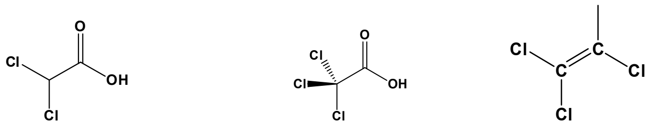

Dichloroacetic acid Trichloroacetic acid Trichloroethylene

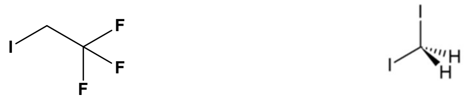

1,1,1-Trifluoro-2-iodoethane Diiodomethane

Scheme 3S. Small organic molecules containing halogen atom(s)

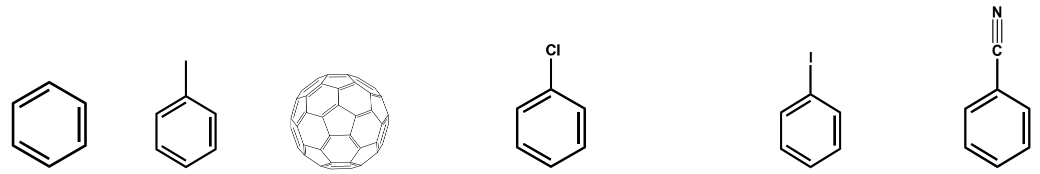

Benzene Toluene Co_{60} Chlorobenzene Iodobenzene Benzonitrile

Scheme 4S. Simple aromatic molecules

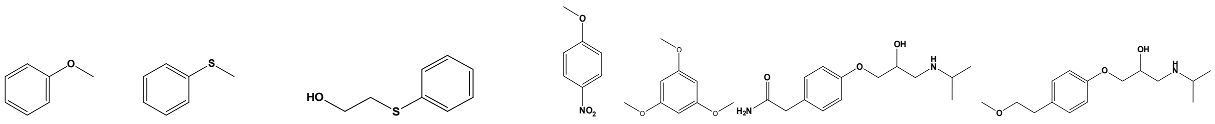

Anisole Thioanisole 2-(Phenylthio)ethanol 4-Nitroanisole TMB Atenolol Metoprolol

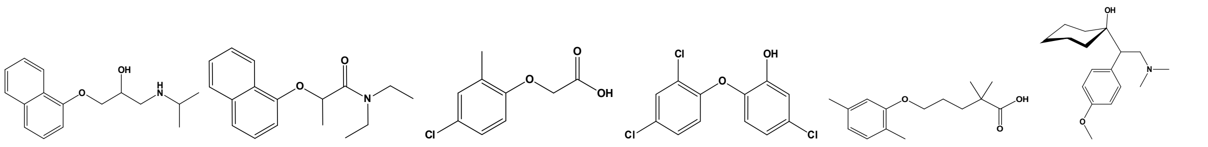

Propranolol Napropamide MCPA Triclosan Gemfibrozil Venlafaxine

Scheme 5S. Anisole and related compounds

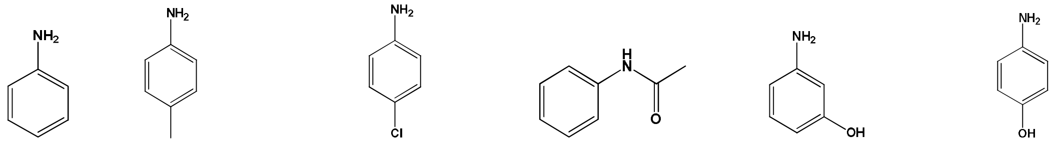

Aniline 4-Toluidine 4-Chloroaniline Acetanilide 3-Amino-phenol 4-Amino-phenol

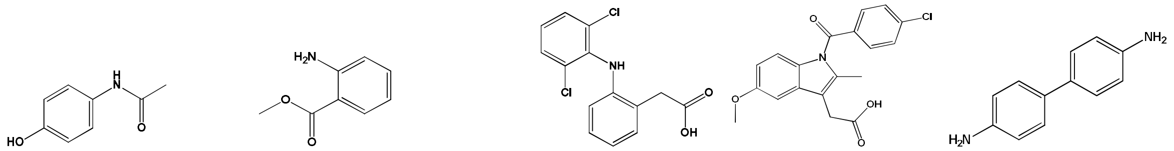

Acetaminophen Methyl-2-aminobenzoate Diclofenac Indomethacin Benzidine

Scheme 6S. Anilines and related compounds

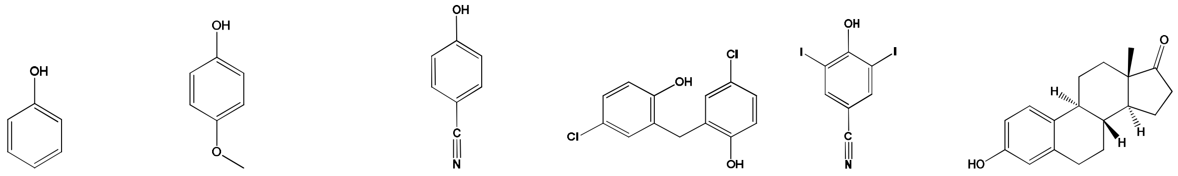

Phenol 4-Methoxyphenol 4-Cyanophenol Dichlorofen Ioxynil Estrone

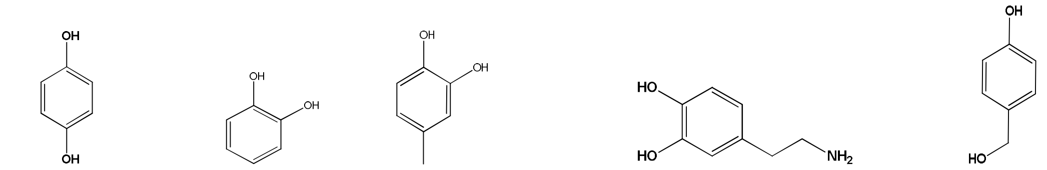

Hydroquinone Catechol 4-Methylcatechol Dopamine 4-Hydroxybenzyl alcohol

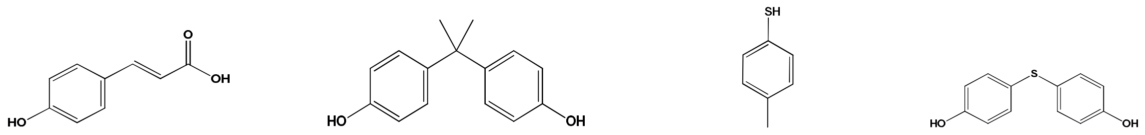

p-Hydroxycinnamic acid Bisphenol A 4-Methylthiophenol 4,4’-Thiodiphenol

Scheme 7S. Phenol type molecules

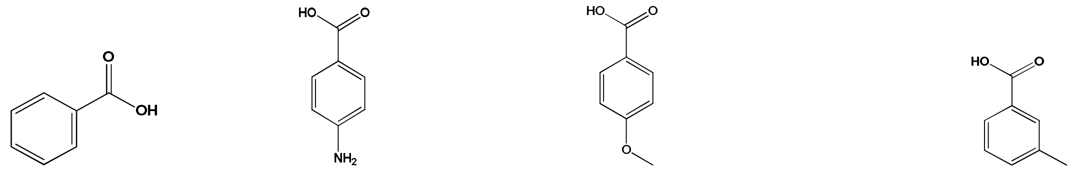

Benzoic acid 4-Aminobenzoic acid 4-Methoxybenzoic acid 3-Methylbenzoic acid

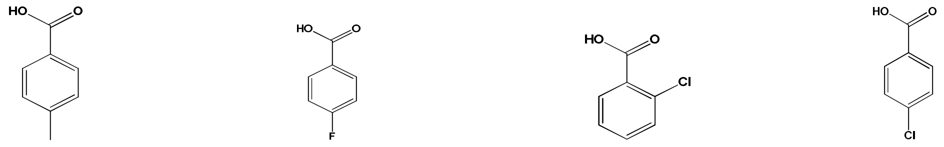

4-Methylbenzoic acid 4-Fluorobenzoic acid 2-Chlorobenzoic acid 4-Chlorobenzoic acid

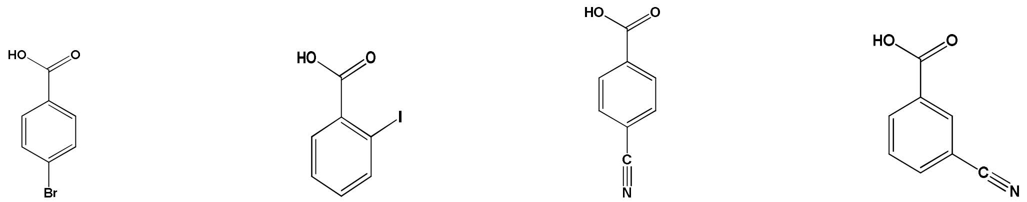

4-Bromobenzoic acid 2-Iodobenzoic acid 4-Cyanobenzoic acid 3-Cyanobenzoic acid

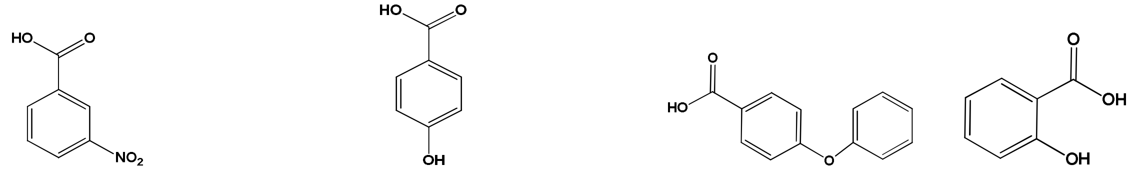

3-Nitrobenzoic acid 4-Hydroxybenzoic acid 4-Phenoxybenzoic acid Salicylic acid

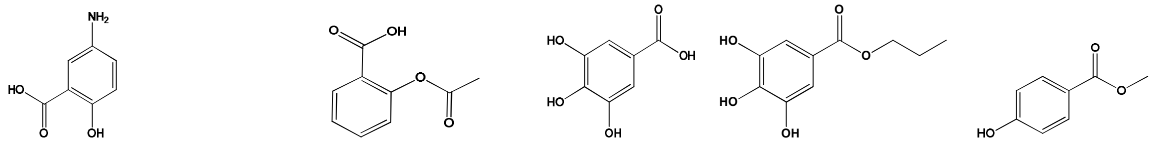

5-Aminosalicylic acid Acetylsalicylic acid Gallic acid Propyl gallate Methyl paraben

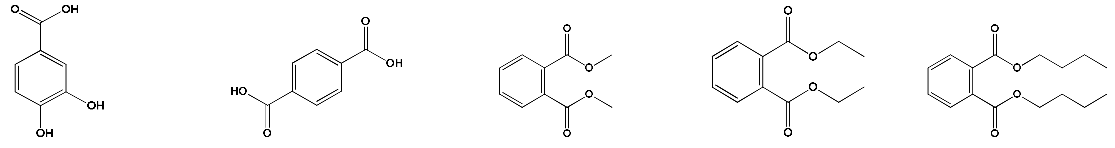

Protocatechuic acid Terephthalic acid Dimethyl phthalate Diethyl phthalate Dibutyl phthalate

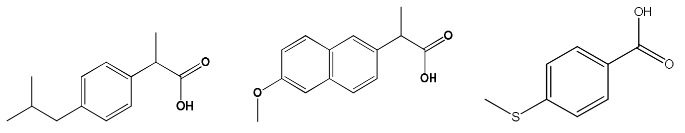

Ibuprofen Naproxen 4-(Methylthio)benzoic acid

Scheme 8S. Benzoic acids and esters

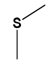 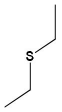 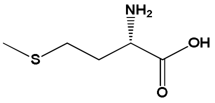 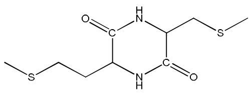 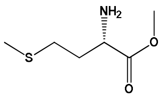

Dimethyl sulfide Diethyl sulfide Methionine Methionine anhydride Methioninemetyl ester

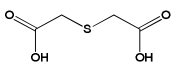 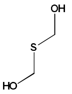 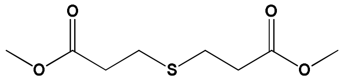 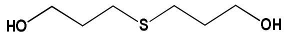

Thiodiglycolic acid 2,2'-Thiodiethanol Dimethyl 3,3’-thiodipropionate 3,3’-Thiodipropionol

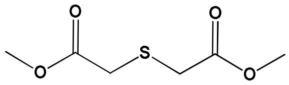 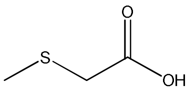 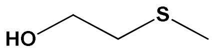

Dimethyl 2,2’-thiodiethanoate Methylthioacetic acid 2-(Methylthio)ethanol

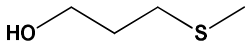 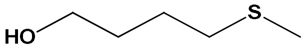 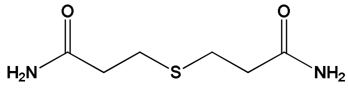

3-(Methylthio)propanol 4-(Methylthio)butanol 3,3’-Thiodipropionamide

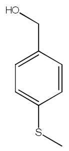 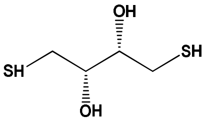 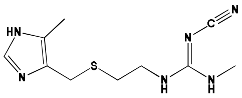

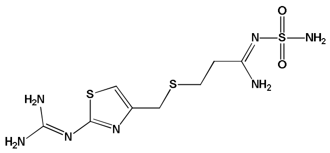

(4-Methylthiophenyl) methanol Dithiothreitol Cimetidine Famotidine

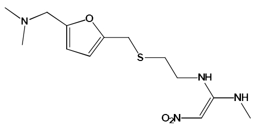 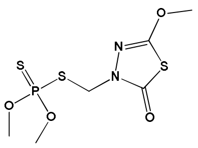 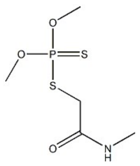 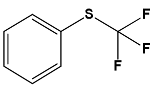 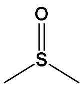 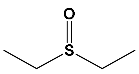

Ranitidine Methidation Dimethoate PTS Dimethyl sulfoxide Diethyl sulfoxide

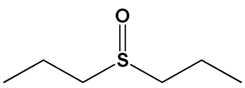 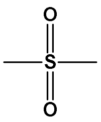 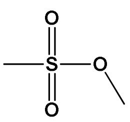 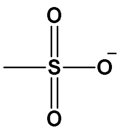 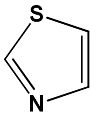

Dipropyl sulfoxide Dimethyl sulfone Methanesulfonate Methanesulfinate Thiazole

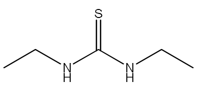 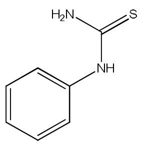 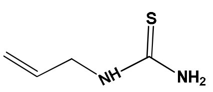 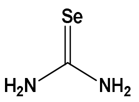 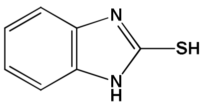

Diethylthiourea Phenylthiourea Allythiourea Selenourea 2-Mercaptobenzimidazole

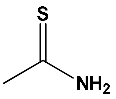 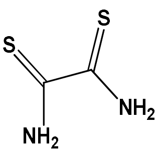 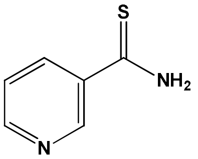 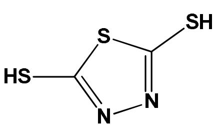

Thioacetamide Dithio-oxamide Thionicotinamide 2,5-Dimercaptothiadiazole

Scheme 9S. Sulfur compounds

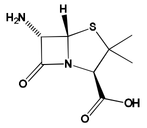 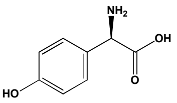 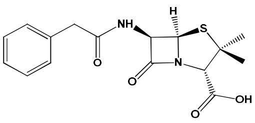 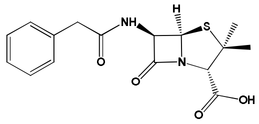

6-Aminopenicillanic acid 4-Hydroxy-D-phenyl glycine Amoxicillin Penicillin G

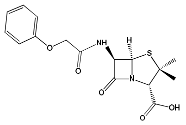 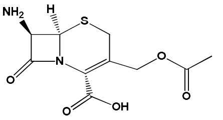 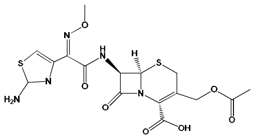 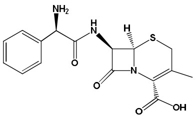 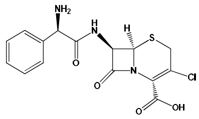

Penicillin V 7-Aminocephalosporanic acid Cefotaxime Cephalexin Cefaclor

Scheme 10S. β-Lactams

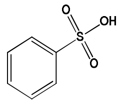 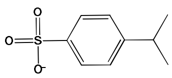 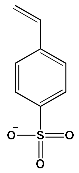 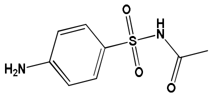 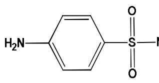

Benzenesulfonic acid *p*-Cumenesulfonate *p*-Styrene sulfonate Sulfacetamide Sulfanilamide

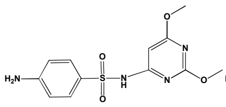 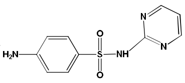 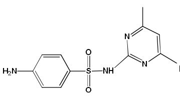 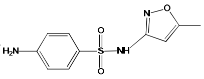 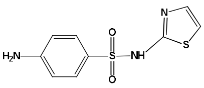 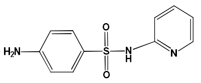

Sulfadimethoxine Sulfadiazine Sulfamethazine Sulfamethoxazole Sulfathiazole Sulfapyridine

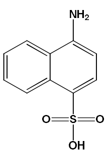 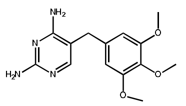

1-Naphthylamine-4-sulfonic acid Trimethoprim

Scheme 11S. Benzenesulfonates

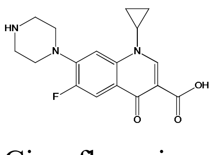 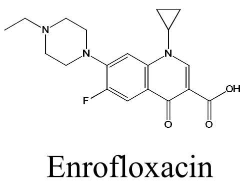 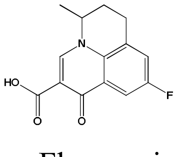 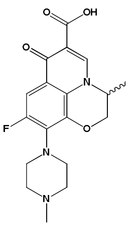

Ciprofloxacin Enrofloxacin Flumequine Ofloxacin

Scheme 12S. Fluoroquinolones

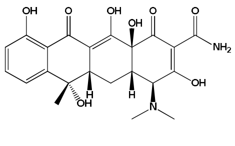 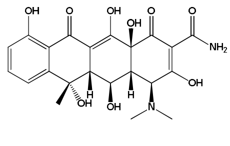 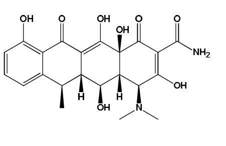 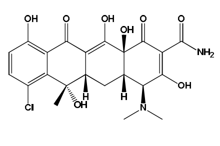 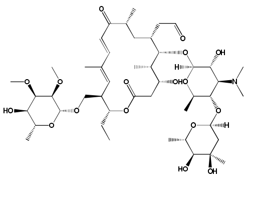

Tetracycline Oxytetracycline Doxycycline Chlorotetracycline Tylosin

Scheme 13S. Tetracycline antibiotics and tylosin

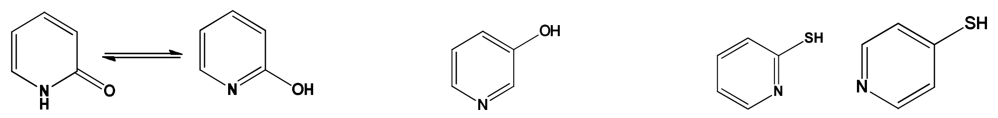

2-Pyridone--2-hydroxy pyridine 3-Hydroxy pyridine 2-Mercaptopyridine 4-Mercaptopyridine

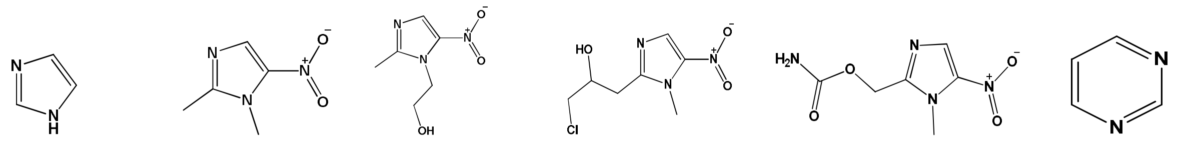

Imidazole Dimetridazole Metronidazole Ornidazole Ronidazole Pyrimidine

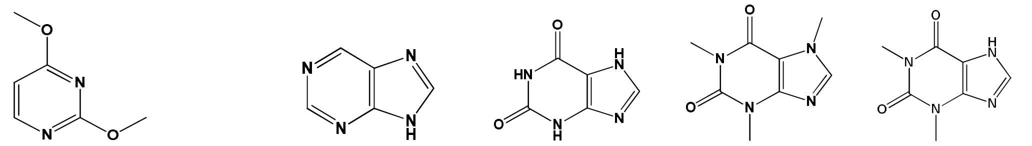

2,4-Dimethoxypyrimidine Purine Xanthine Caffeine Theophylline

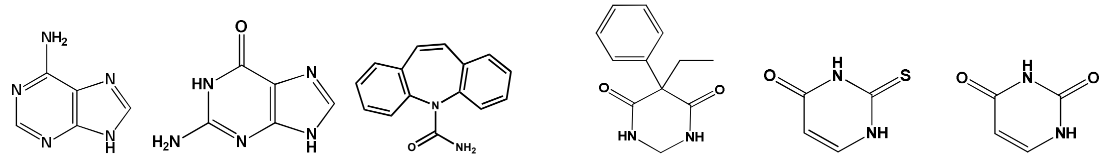

Adenine Guanine Carbamazepine Pirimidone 2-Thiouracil 4-Thiouracil

Scheme 14S. Molecules with nitrogen atom(s) in the ring

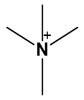 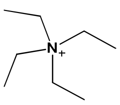 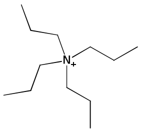

Tetramethylammonium cation Tetraethylammonium cation Tetrapropylammonium cation

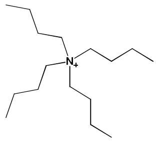 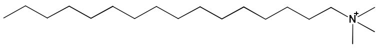

Tetrabutylammonium cation Cetyltrimethylammonium cation

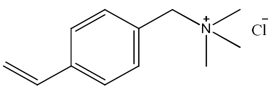 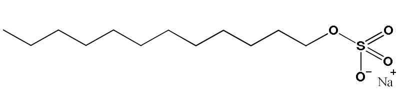 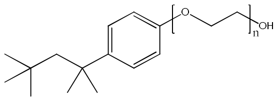

Vinylbenzyltrimethylammonium chloride Sodium dodecyl sulfate Igepal CO-730

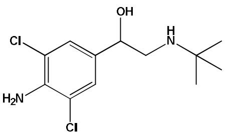 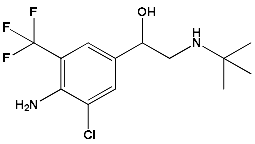 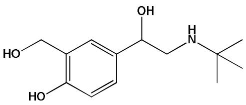 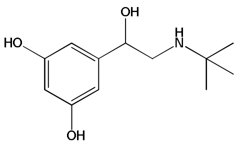

Clenbuterol Mabuterol Salbutamol Terbutaline

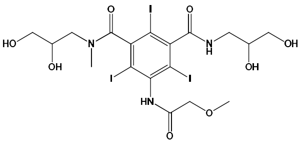 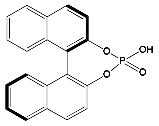 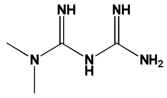 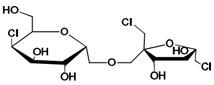 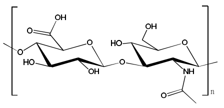

Iopromide $BiNPO_4H$ Metformin Sucralose Hyaluronan

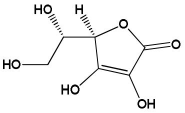 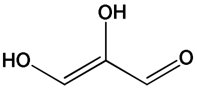 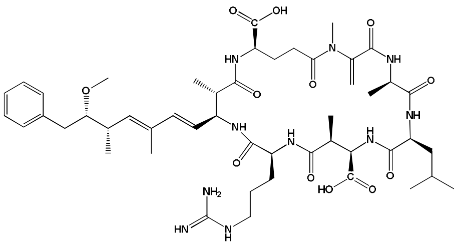

Ascorbic acid 2,3-Dihydroxy-2-propenal Microcystin-LR

Scheme 15S. Miscellaneous compounds.

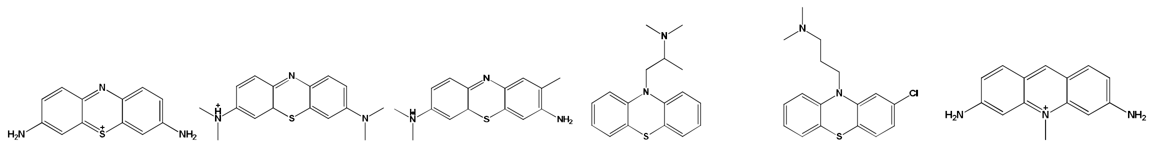

Thionine Methylene blue Toluidine blue Promethazine Chlorpromazine Acriflavine cation

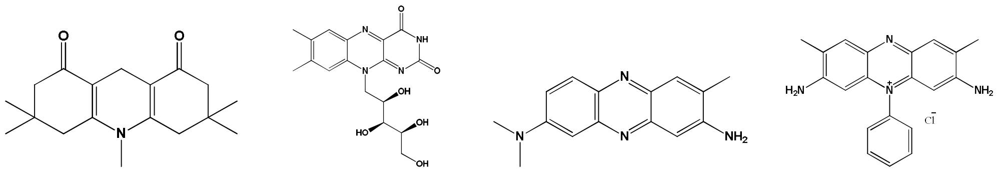

Acridine-1,8-dione Riboflavine Neutral red Safranine T cation

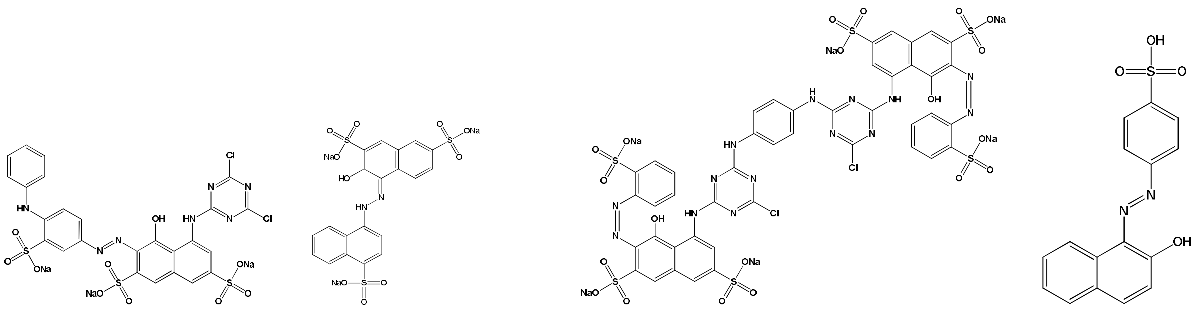

Reactive Blue 81 Acid Red 27 Reactive Red-120 dye Orange II

Scheme 16S. Dyes

References

Armstrong DA, Sun Q, Schuler RH (1996) Reduction potentials and kinetics of electron transfer reactions of phenylthiyl radicals: comparison with phenoxyl radicals. J Phys Chem 100:9892-9899. https://doi.org/10.1021/jp960165n

Arnold WA (2014) One electron oxidation potential as a predictor of rate constants of N-containing compounds with carbonate radical and triplet excited state organic matter. Environ Sci: Processes Impacts 16:832-838. https://doi.org/10.1039/C3EM00479A

Canonica S, Kohn T, Mac M, Real FJ, Wirz J, von Gunten U (2005) Photosensitizer method to determine rate constants for the reaction of carbonate radical with organic compounds. Environ Sci Technol 39:9182-9188. https://doi.org/10.1021/es051236b

Dhiman SB, Naik DB (2010) Nature of the transient species formed in the pulse radiolysis of 4-hydroxybenzyl alcohol in aqueous solutions: observation of equilibrium in the reaction of OH-adducts with HPO_4^{2-} ions. J Phys Org Chem 23:48-55. https://doi.org/10.1002/poc.1583

Elango TP, Ramakrishnan V, Vancheesan S, Kuriacose JC (1984) Reaction of the carbonate radical with substituted anilines. Proc Indian Academy Sci, Chem Sci 93:47-52. https://doi.org/10.1007/BF02841982

Geeta S, Sharma SB, Rao BMS, Mohan H, Dhanya S, Mittal JP (2001) Study of kinetics and absorption spectra of OH adducts of hydroxy derivatives of benzaldehyde and acetophenone. J Photochem Photobiol A: Chem 140:99-107. https://doi.org/10.1016/S1010-6030(01)00402-6

Hasegawa K, Neta P (1978) Rate constants and mechanisms of reaction of Cl_2^{-}. J Phys Chem 82:854-857. https://doi.org/10.1021/j100497a003

Huang J, Mabury SA (2000) A new method for measuring carbonate radical reactivity toward pesticides. Environ Toxicol Chem 19:1501-1507. https://doi.org/10.1002/etc.5620190605

Huang Y, Kong M, Westerman D, Xu EG, Coffin S, Cochran KH, Liu Y, Richardson SD, Schlenk D, Dionysiou DD (2018) Effects of HCO_3^{-} on degradation of toxic contaminants of emerging concern by UV/NO_3^{-}. Environ Sci Technol 52:12697-12707. https://doi.org/10.1021/acs.est.8b04383

Huie RE, Shoute LCT, Neta P (1991) Temperature dependence of the rate constants for reactions of the carbonate radical with organic and inorganic reductants. International J Chem Kinet 23:541-552. https://doi.org/10.1002/kin.550230606

Jonsson M, Lind J, Reitberger T, Eriksen TE, Merényi G (1994) Redox and acidity properties of 4-substituted aniline radical cations in water. J Amer Chem Soc 116:1423-1427. https://doi.org/10.1021/ja00083a030

Jovanovic SV, Tosic M, Simic MG (1991) Use of the Hammett correlation and σ^{+} for calculation of one-electron redox potentials of antioxidants. J Phys Chem 95:10824-10827. https://doi.org/10.1021/j100179a054

Kishore K, Moorthy PN, Guha SN (1991) Pulse radiolysis study of one electron oxidation of riboflavin. Radiat Phys Chem 38:119-125. https://doi.org/10.1016/1359-0197(91)90053-5

Lei Y, Cheng S, Luo N, Yang X, An T (2019) Rate constants and of mechanisms for the reactions of $Cl^{\bullet}$ and $Cl_2^{\bullet-}$ with trace organic contaminants. Environ Sci Technol 53:11170-11182. https://doi.org/10.1021/acs.est.9b02462

Li C, Hoffman MZ (1999) One-electron redox potentials in aqueous solution. J Phys Chem B 203:6653-6656. https://doi.org/10.1021/jp983819w

Lind J, Shen X, Eriksen TE, Merényi G (1990) The one-electron reduction potential of 4-substituted phenoxyl radicals in water. J Amer Chem Soc 112:479-482. https://doi.org/10.1021/ja00158a002

Marcus RA (1965) On the theory of electron transfer reactions VI. United treatment for homogeneous and electrode reactions. J Chem Phys 43:679-701. https://doi.org/10.1063/1.1696792

Marcus RA, Sutin N (1985) Electron transfer in chemistry and biology. Biochimica et Biophysica Acta (BBA) – Rev Bioenerg 811:265-322. https://doi.org/10.1016/0304-4173(86)90003-0

Meisel D (1975) Free energy correlation of rate constants for electron transfer between organic systems in aqueous solution. Chem Phys Lett 34:263-266. https://doi.org/10.1016/0009-2614(75)85269-9

Moore JS, Phillips GO, Sosnowski A (1977) Reaction of the carbonate radical anion with substituted phenols. Int J Radiat Biol 31:603-605. https://doi.org/10.1080/09553007714550691

Neta P, Madhavan V, Zemel H, Fessenden RW (1977) Rate Constants and mechanism of reaction of SO_4^- with aromatic compounds. J Am Chem Soc 99:163-164. https://doi.org/10.1021/ja00443a030

Pavitt AS, Bylaska EJ, Tratnyek PG (2017) Oxidation potentials of phenols and anilines: correlation analysis of electrochemical and theoretical values. Environ Sci: Process Impacts 19:339-349. https://doi.org/10.1039/C6EM00694A

Roder M, Földiák G, Wojnárovits L (1999) Electron transfer from cresols to $N_3^•$, $BrO_2^•$, $ClO_2^•$, $NO_2^•$ and $SO_4^{•-}$ radicals: correlation between rate constants and one-electron reduction potentials. Radiat Phys Chem 55:515-519. https://doi.org/10.1016/S0969-806X(99)00240-6

Sataraddi SR, Patil SM, Bagoji AM, Pattar VP, Nandibewoor ST (2014) Electrooxidation of indomethacin at multiwalled carbon nanotubes-modified GCE and its determination in pharmaceutical dosage form and human fiological fluids. ISRN Anal Chem, No. 816012, http://dx.doi.org/10.1155/2014/816012

Schwarz HA, Comstock D, Yandell JK, Dodson RW (1974) Pulse radiolysis study of thallium(II) in aqueous perchloric acid solutions. J Phys Chem 78:488-493. https://doi.org/10.1021/j100598a005

Steenken S, Neta P (1982) One-electron redox potentials of phenols. Hydroxy- and aminophenols and related compounds of biological interest. J Phys Chem 86:3661-3667. https://doi.org/10.1021/j100215a033

Stenman D, Carlsson M, Jonsson M, Reitberger T (2003) Reactivity of the carbonate radical anion towards carbohydrate and lignin model compounds. J Wood Chem Technol 23:47-69. https://doi.org/10.1081/WCT-120018615

Thu NTA, Van Duc H, Hai Phong NH, Cuong ND, Hoan NTV, Khieu DQ (2018) Electrochemical determination of paracetamol using Fe_3O_4/reduced graphene-oxide-based electrode. J Nanomaters No. 7619419, https://doi.org/10.1155/2018/7619419

Wardman P (1989) Reduction potentials of one-electron couples involving free radicals in aqueous solution. J Phys Chem Ref Data 18:1637-1755. https://doi.org/10.1063/1.555843

Wojnárovits L, Takács E (2019) Rate constants of sulfate radical anion reactions with organic molecules: a review. Chemosphere 220:1014-1032. https://doi.org/10.1016/j.chemosphere.2018.12.156

Wojnárovits L, Tóth T, Takács E (2020) Rate constants of carbonate radical anion reactions with molecules of environmental interest in aqueous solution: a review. Sci Total Environ No. 137219; https://doi.org/10.1016/j.scitotenv.2020.137219
